# *Candida albicans* Multilocus Sequence Typing Clade I Contributes to the Clinical Phenotype of Vulvovaginal Candidiasis Patients

**DOI:** 10.3389/fmed.2022.837536

**Published:** 2022-04-01

**Authors:** Yuxia Zhu, Chao Fang, Yu Shi, Yingying Shan, Xiaoping Liu, Yiheng Liang, Liting Huang, Xinyang Liu, Chunfeng Liu, Yin Zhao, Shangrong Fan, Xiaowei Zhang

**Affiliations:** ^1^Department of Obstetrics and Gynecology, Peking University Shenzhen Hospital, Shenzhen, China; ^2^Shenzhen PKU-HKUST Medical Center, Institute of Obstetrics and Gynecology, Shenzhen, China; ^3^Shenzhen Key Laboratory on Technology for Early Diagnosis of Major Gynecological Disease, Shenzhen, China; ^4^BGI-Shenzhen, Shenzhen, China; ^5^Clinical College of Peking University Shenzhen Hospital, Anhui Medical University, Hefei, China; ^6^Department of Laboratory Science, Peking University Shenzhen Hospital, Shenzhen, China; ^7^Research Institute of Huazhong University of Science and Technology in Shenzhen, Shenzhen, China

**Keywords:** multilocus sequence typing, *Candida albicans*, clinical characterization, antifungal resistance, virulence

## Abstract

*Candida albicans* is the most frequent fungal species responsible for vulvovaginal candidiasis (VVC), which exhibits distinct genetic diversity that is linked with the clinical phenotype. This study aimed to assess the genotypes and clinical characteristics of different *C. albicans* isolates from VVC patients. Based on multilocus sequence typing (MLST), clade 1 was identified as the largest *C. albicans* group, which appeared most frequently in recurrent VVC and treatment failure cases. Further study of antifungal susceptibility demonstrated that MLST clade 1 strains presented significantly higher drug resistance ability than non-clade 1 strains, which result from the overexpression of MDR1. The mRNA and protein expression levels of virulence-related genes were also significantly higher in clade 1 isolates than in non-clade 1 isolates. Proteomic analysis indicated that the protein stabilization pathway was significantly enriched in clade 1 strains and that RPS4 was a central regulator of proteins involved in stress resistance, adherence, and DNA repair, which all contribute to the resistance and virulence of MLST clade 1 strains. This study was the first attempt to compare the correlation mechanisms between *C. albicans* MLST clade 1 and non-clade 1 strains and the clinical phenotype, which is of great significance for VVC classification and treatment.

## 1. Introduction

Nearly three-quarters of healthy women experience at least one episode of vulvovaginal candidiasis (VVC) during their lifetimes, and 5–10% of women suffer from recurrent VVC (RVVC), defined as at least three episodes of VVC within 1 year (1). The high recurrence rate has been a difficult clinical problem for decades due to various complex factors (2). Invasive fungal pathogens, the root etiology of VVC, harbor different genotypes that lead to varying degrees of host immune responses and further determine different infection outcomes, antifungal drug efficacy and recurrence probability. *Candida albicans* is the most frequent fungal species responsible for VVC and exhibits distinct phenotypic and genetic diversity (3). The genotype distribution of *C. albicans* strains has been associated with different clinical conditions of VVC (4, 5). The genotypic differences of *C. albicans* strains retrieved from VVC patients and healthy women reveal that VVC-linked strains possess specific genotypes (6). Hence, studying the genotypes of different subspecies of *C. albicans* strains will contribute to understanding their pathogenic mechanisms, which is important for increasing the recovery rate and controlling the infection rate.

Different molecular methods have been applied to identify the genotypes of *C. albicans* strains, including non-DNA-based methods, DNA-based conventional typing methods and exact DNA-based techniques (7). Multilocus enzyme electrophoresis (MLEE), a classic non-DNA-based method, has been replaced by conventional DNA-based typing methods with higher resolution, including electrophoretic karyotyping (EK), random amplified polymorphic DNA (RAPD), restriction fragment length polymorphism (RFLP), and repetitive extragenic palindromic element PCR (REP-PCR) (3). Southern blot hybridization with the DNA fingerprinting probe Ca3 is a widely used technique for the exact typing of *C. albicans* isolates, but the experimental process is particularly complex, so it was replaced by microsatellite length polymorphism (MLMT) and multilocus sequence typing (MLST). The MLST method directly analyzes polymorphisms within seven housekeeping DNA markers and exhibits high discriminatory power and reproducibility (8). To date, a public database for MLST data for *C. albicans* has been established (http://calbicans.mlst.net) that can be used to evaluate the worldwide diversity of *C. albicans*. The population structure of *C. albicans* consists of 18 well-defined clades (9) and is associated with epidemiological characteristics, such as the infection's anatomical location, geographical distribution, and antifungal drug susceptibility (10, 11). However, new clades have been successively identified, with an increase in the number of isolates from the baseline, which has also resulted in a more diversified association. Therefore, additional isolates with detailed epidemiological characteristics are needed to provide a more accurate correlation between the genotype of *C. albicans* and the phenotype of VVC, which will be helpful for guiding clinical diagnosis and treatment.

The drug resistance and virulence factors of the strain are also closely associated with the clinical characteristics of VVC, which can be attributed to the phenotypic plasticity and high genetic diversity of strains. A previous study indicated that the reduction in the 5-fluorocytosine susceptibility of MLST clade 1 strains was linked to mutation of the FUR1 gene (12), and isolates with significantly lower levels of acid phosphatase activity were clustered in MLST clade 2, which resulted in a higher virulence phenotype of the strains, as well as a higher number of tandem repeat sequences in the hyphal regulator genes (HYR1 and HYR3) and the agglutinin-like sequence (ALS) cell surface protein-encoding genes (13). Therefore, the phenetic and genetic profile related to the fungal susceptibility and virulence properties of clinical *C. albicans* isolates is necessary for optimizing the treatment methods for VVC and controlling the transmission of drug resistance. Furthermore, comparative proteomic studies have been performed to explain the mechanism of drug resistance in untreated control cells and cells treated with antifungal drugs (14–16), as well as avirulent and virulent *C. albicans* strains (17). However, the proteomic profiles among different MLST genotype clusters remain unclear.

In the current study, we aimed to assess the relevance of the association between the clinical characteristics and the MLST phenotype of *C. albicans* isolates from VVC patients and explain the MLST clade-specific antifungal drug resistance and virulence properties. This information will greatly support the graded diagnosis and treatment strategy of VVC.

## 2. Materials and Methods

### 2.1. The Isolation of *C. albicans* Strains

This study was approved by the Medical Review Board of Peking University Shenzhen Hospital. All *C. albicans* were isolated from vaginal samples of VVC patients in the Department of Obstetrics and Gynecology, Peking University Shenzhen Hospital, between April 2003 and December 2016. All the clinical information of the participants, including age, pregnancy status, and treatment history and results, was recorded after a consent form was signed.

Vaginal samples from the lateral vaginal wall were collected with a sterile cotton-tipped swab. The swab was placed in a tube filled with saline for microscopic examination with 10% potassium hydroxide. Specimens were simultaneously inoculated on Sabouraud's dextrose agar (SDA) plates and CHROMagar Candida plates (Biocell Laboratory Ltd., Zhengzhou, China) for 24–48 h at 37°C in ambient air. Single colonies obtained from the SDA plates were identified using the standardized Candida API system (bioMerieux, Marcy l'Etoile, France) and stored in medium containing 2% glucose, 2% peptone, and 20% glycerol at −70°C.

### 2.2. DNA Extraction and MLST Genotyping of the Isolates

The DNA of the isolates was extracted by an EZNA TM Yeast DNA Kit (Omega Bio-Tek, USA) according to the manufacturer's instructions. For MLST identification, seven housekeeping genes (AAT1a, ACC1, ADP1, MPIb, SYA1, VPS13, and ZWF1b) were amplified and sequenced. The primers are shown in Table 1. The sequences were uploaded to the *C. albicans* MLST database (http://calbicans.mlst.net) for genotype identification. Then, DNA sequences of the seven marker genes were concatenated for cluster analysis and clade definition, and a phylogenetic tree was constructed by the unweighted-pair group method using average linkages (UPGMA) according to the p-distance model (*p* = 0.04) with 1,000 replicates for bootstrap testing (MEGA 6.0). Additionally, we identified the ABC genotype and mating type locus (MTL) homozygosity of each isolate according to previously described methods (18, 19).

**Table 1:** Primers used in this study.

**Fragment**	**Forward (5^**′**^-3^**′**^)**	**Reverse (5^**′**^-3^**′**^)**	**References**
AAT1a	ACTCAAGCTAGATTTTTGGC	CAGCAACATGATTAGCCC	(20)
ACC1	GCAAGAGAAATTTTAATTCAATG	TTCATCAACATCATCCAAGTG	(20)
ADP1	GAGCCAAGTATGAATGATTTG	TTGATCAACAAACCCGATAAT	(20)
MPIb	ACCAGAAATGGCCATTGC	GCAGCCATGCATTCAATTAT	(20)
SYA1	AGAAGAATTGTTGCTGTTACTG	GTTACCTTTACCACCAGCTTT	(20)
VPS13	TCGTTGAGAGATATTCGACTT	ACGGATGGATCTCCAGTCC	(20)
ZWF1b	GTTTCATTTGATCCTGAAGC	GCCATTGATAAGTACCTGGAT	(20)
MTLa	AGAGGCAGGAGAACAGCAAC	TCCAACGCAAACTCTTCGG	(21)
MTLα	CGTCTTCATCAATGGTGTCTGC	TCAACGCTTGCTGTCTCACA	(21)
ACT1	TGCTGAACGTATGCAAAAGG	TGAACAATGGATGGACCAGA	(22)
SAP1	TGAGGCTGCTGGTGATTATG	TGCCAACAGCTTTGAGAGAA	(23)
SAP2	ATCAGCTGGTTTCGTTGCTT	GGGACAGCTTGTCTTTTGGA	(23)
SAP3	TGTTACTGGTCCCCAAGGTGAA	CTTGTCCTTGACCAGCTTGACAT	(23)
SAP4	AATGATGTGGGCAAAAGAGG	ACGGCATTTGAATCTGGAAC	(23)
SAP5	ATTTCCCGTCGATGAGACTG	ACCACGCCATTTTGGAATAC	(23)
SAP6	GTCAACGCTGGTGTCCTCTT	GCAGGAACGGAGATCTTGAG	(23)
SAP7	TTCTCGTGATGCTGTCCAAG	CCAGCAGGAAGACCATAAGC	(23)
SAP8	TTTGGTGGGGTTGATAATGC	GGCAGCAGCCAATTTATCAG	(23)
SAP9	ACCGGGTCTTCAGATTTGTG	TTCCTCGTCGGTTTCTATGG	(23)
SAP10	AACGGAAATGTTGCTTCTGG	TGAATCGCCTATCGAAAACC	(23)
PLB1	GCTCTTTTCAACGAAGCGGTGT	GCCATCTTCTCCACCGTCAACT	(22)
PLB2	CAATACTAGCCGCGTTGGGAAG	GCCCATGAAAAACCCTGCATTA	(22)
PLB3	TCCCAATTGTTGTTGCTGATGG	CCGCATTATCAAACCCACCAAT	(22)
PLB5	TCGTCCGGGTCTTCAAGTTCTC	ATCTCCCGAATCCCCGTCTAAA	(22)
ALS1	CCCAACTTGGAATGCTGTTT	TTTCAAAGCGTCGTTCACAG	(22)
ALS2	GCACTTCATTGACTGGAGCA	TCATTGTTGCCACCTTGTGT	(22)
ALS3	CTGGACCACCAGGAAACACT	GGTGGAGCGGTGACAGTAGT	(22)
ALS4	TCCACAGTTTCTCGTCCACA	ATTGCCACGCTTGTTTTACC	(22)
ALS5	GTTCAGACATGCCATCATCG	CCAAGTGATCAGGGTGGACT	(22)
ALS6	ATCGGAAGCTCCAAATTCCT	AGGATGTTTAGTGGCGGATG	(22)
ALS7	GACCTTTTGTGGATGCGATT	TTTTCTGGAGTCGGGAAATG	(22)
ALS9	CCATATTCAGAAACAAAGGGTTC	AACTGAAACTGCTGGATTTGG	(24)
HWP1	TCTACTGCTCCAGCCACTGA	CCAGCAGGAATTGTTTCCAT	(22)
CDR1	TTTAGCCAGAACTTTCACTCATGATT	TATTTATTTCTTCATGTTCATATGGATTGA	(25)
CDR2	GGTATTGGCTGGTCCTAATGTGA	GCTTGAATCAAATAAGTGAATGGATTAC	(25)
MDR1	TTACCTGAAACTTTTGGCAAAACA	ACTTGTGATTCTGTCGTTACCG	(25)
ERG11	AACTACTTTTGTTTATAATTTAAGA TGGACTATTGA	AATGATTTCTGCTGGTTCAGTAGGT	(25)

### 2.3. RNA Extraction and Quantitative Real-Time PCR

The RNA of the isolates was extracted by an EZNA TM Yeast RNA Kit (Omega Bio-Tek, USA) and reverse transcribed to cDNA using a Takara PrimeScriptRT Reagent Kit with gDNA Eraser (Takara, Japan) according to the manufacturer's instructions. Quantitative real-time PCR analysis of the genes related to drug resistance, including CDR1, CDR2, MDR1, and ERG11, and virulence factors, including ALS1-7, ALS9, HWP1, SAP1-SAP10, PLB1-3, and PLB5, was performed as described previously (22, 23, 25). The ACT1 gene was used as a housekeeping gene. Data for each target gene were calculated as the fold change in comparison with the reference gene ACT1 using the ΔΔCt quantification method. The primers used for quantitative real-time PCR analysis are shown in Table 1.

### 2.4. Antifungal Susceptibility Testing

The susceptibilities of the isolates to antifungal drugs were assayed by using the broth microdilution method according to the proposed Clinical and Laboratory Standards Institute M27-A3 and M60 standard guidelines (26, 27). In total, 14 antifungal drugs were selected for susceptibility testing, and they were grouped into five categories: imidazoles, triazoles, polyenes, echinocandins, and others. The fat-soluble drugs, including itraconazole (ITC), miconazole (MCA), clotrimazole (CLO), voriconazole (VRC), terconazole (TEC), butoconazole (BUC), amphotericin B (AMB), terbinafine (TRB), caspofungin (CFG), micafungin (MFG), and anidulafungin (AFG), were dissolved in DMSO, and the storage concentration was at least 100 times greater than the test concentration to avoid the influence of DMSO. Water-soluble drugs, including fluconazole (FLC), nystatin (NYS), and 5-fluorocytosine (5FC), were dissolved in sterile normal saline solution. The test concentration range of FLC, NYS, and 5FC was 0.125–64 μg/ml, and their storage concentration was 1,280 μg/ml. The test concentration range of ITC, MCA, CLO, VRC, TEC, BUC, AMB, and TRB was 0.0313–16 μg/ml, and their storage concentration was 3,200 μg/ml. The test concentration range of CFG, MFG, and AFG was 0.015–8 μg/ml, and their storage concentration was 1,600 μg/ml. For susceptibility testing, 5 single colonies with a diameter of ~1 mm were picked and suspended in 5 ml of sterile normal saline solution. After vortexing, the bacterial suspension was diluted to 1 × 10^3^ CFU/ml −5 × 10^3^ CFU/ml with RPMI1640 medium. Then, a mixture of 100 μl bacterial suspension and 100 μl drug solution was added to 96-well plates and incubated at 35°C. The minimum inhibitory concentration (MIC) values for AMB and NYS were determined as the minimum concentrations required for completely clear culture after 48 h of incubation. The MIC values for imidazoles, triazoles, and 5FC were determined as the minimum concentrations required for the culture to have a 50% turbidity decrease after 48 h of incubation. The MIC values for echinocandins were determined as the minimum concentrations required for the culture to have a 50% turbidity decrease after 24 h of incubation. The MIC of each isolate was recorded, and MIC90, which is the minimum inhibitory concentration required to inhibit the growth of 90% of isolates in each clade, was calculated.

### 2.5. Extracellular Enzyme Assays

The production of extracellular enzymes, such as esterase, hemolysis enzyme, and phospholipase, was measured to reflect the virulence of the strains. The esterase activity and hemolysis enzyme production were evaluated using the plate assays described by Pakshir et al. (28), while the phospholipase activity was determined using base medium modified by adding 0.15% potassium tellurite to the egg yolk emulsion to improve the visualization of the halos of precipitation and the accuracy of the measurements (29).

### 2.6. Quantitative Proteomic Analysis

In total, six strains were randomly selected for the proteomic study, including three strains from MLST clade 1 (2 isolates in this study and the type strain *C. albicans* ATCC90028) and three non-clade 1 strains. Proteomic analysis was carried out using liquid chromatography-tandem mass spectrometry (LC-MS/MS; SWATH-MS) by Wuhan GeneCreate Biological Engineering Co., Ltd, China (http://www.genecreate.com/). Total proteins were extracted using the ultrasonic crushing method (30). An AB SCIEX nano LC-MS/MS (Triple TOF 5600 plus) system was used for liquid chromatography-electrospray ionization-tandem mass spectrometry (LC-ESI-MS/MS) analysis. Spectral library generation and SWATH data processing were performed using Skyline version 2.5 software (Department of Genome Sciences, University of Washington). The interactions among proteins with significantly different abundances were analyzed and visualized by Cytoscape (version 3.8.2).

### 2.7. Statistical Analysis

The statistical significance of the results was determined by the Wilcoxon test. The results were considered statistically significant when the *p-values* were <0.05.

## 3. Results

### 3.1. MLST Clade 1 Dominates the *C. albicans* Isolate Genotype Distribution

**Figure 1:**
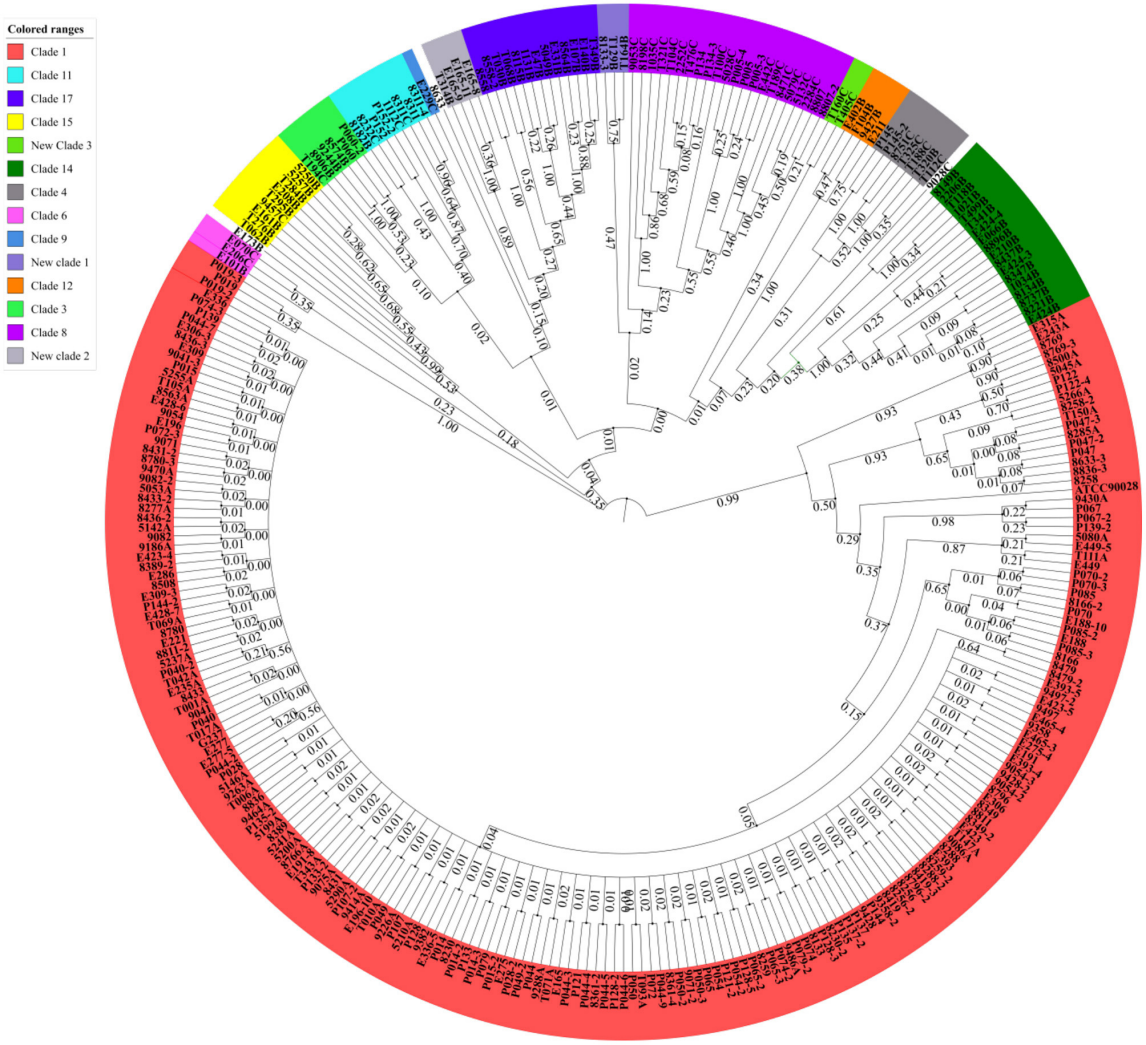
*Candida albicans* isolated from VVC patients were mainly clustered in MLST clade 1. The MLST phylogenetic tree was constructed by UPGMA according to the p-distance model (*p* = 0.04) with 1,000 replicates for bootstrap testing. In total, 306 strains were clustered into 14 clades and three singletons, including 11 known clades and three novel clades. Clade 1 was the largest group, accounting for 66% of the total isolates.

A total of 306 *C. albicans* isolates were analyzed by MLST (Supplementary Table 1). Single nucleotide polymorphisms (SNPs) of housekeeping genes were found after alignment, and the VPS13 and MPIb genes showed the highest rates of variation. Based on MLST database annotation, 76 diploid sequence types (DSTs) were identified, of which DST79 and DST1867 were the most common, including 79 strains and 71 strains, respectively. Moreover, 28 novel DSTs (DST3115-DST3148) were discovered. A dendrogram demonstrated that the 306 *C. albicans* isolates were clustered into 14 clades, including 11 known clades reported by Odds et al. (31) and 3 novel clades (Figure 1). Clade 1 was the largest group, accounting for 66% of the total isolates (202/306), followed by clade 8 (22/306), clade 14 (18/306), clade 17 (13/306), clade 15 (9/306), clade 11 (8/306), clade 4 (7/306), clade 3 (6/306), clade 12 (4/306), clade 6 (3/306), and clade 9 (1/306). The three novel clades discovered in this study were formed by three strains (E173B, 9028C, and 8633), which each constituted a single clade and were designated clade 101, clade 102, and clade 103, respectively. In addition, type A and heterozygotes were the main groups identified by ABC and MTL genotyping, respectively (Supplementary Table 1).

### 3.2. Worse Clinical Presentation of VVC Associated With *C. albicans* MLST Clade 1 Strains

**Figure 2:**
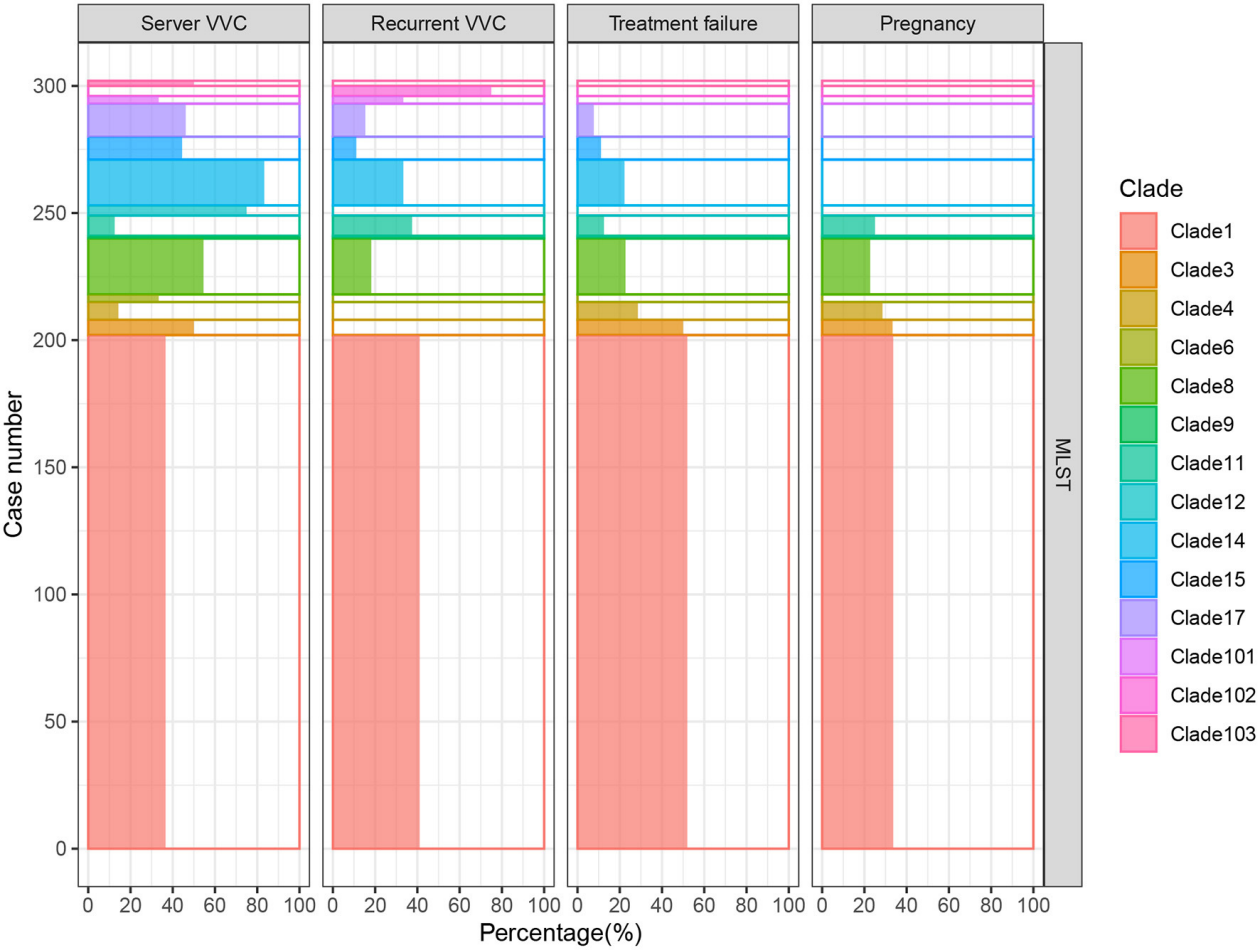
RVVC cases and VVC cases with treatment failure were mainly caused by *C. albicans* in MLST clade 1. In total, 306 samples were analyzed, and the 14 detected clades are presented in different colors. The percentage of clinical characteristics, including severe VVC, RVVC, treatment failure, and pregnancy cases, in each group is shown on the X axis. Clade 1 was dominant in RVVC and treatment failure cases, which demonstrated that VVC caused by invasive clade 1 *C. albicans* was more difficult to cure and relapsed more easily. Although isolates of clade 14 accounted for the highest proportion of severe VVC cases, the cure rate reached 77.8% (14/18). This result revealed that the strains in clade 14 had higher virulence but lower drug resistance.

A total of 306 *C. albicans* strains were isolated from VVC patients with different clinical characteristics, and the relationship between the clinical characteristics and strains is shown in Figure 2. Among the strains, 79 were from pregnant patients, and they were mostly from MLST clades 1, 3, 4, 8, and 9; this distribution was not significantly different from that of strains from nonpregnant patients. In addition, isolates from MLST clade 1 occurred most frequently in RVVC cases and treatment failure cases, as well as genotype A isolates (Supplementary Figure 1). Moreover, for the 44 treatment failure cases, the strains isolated from their subsequent samples were all identified as having the same MLST genotype, which mostly belonged to clade 1 (Supplementary Table 2). These results indicated that VVC caused by invasive *C. albicans* belonging to clade 1 was more difficult to cure and more likely to relapse than VVC caused by non-clade 1 *C. albicans*. In addition, the severity of VVC symptoms at the time of the patient's visit was diagnosed according to a published Chinese specification (32) and recorded. Although severe cases accounted for the highest percentage within MLST clade 14, the cure rate reached 77.8% (14/18). This result revealed that the strains in clade 14 had higher virulence but lower drug resistance. The same phenotype was present in genotype B and homozygous isolates (Supplementary Figure 1).

### 3.3. Lower Antifungal Susceptibility and Gene/Protein Expression in MLST Clade 1

**Figure 3:**
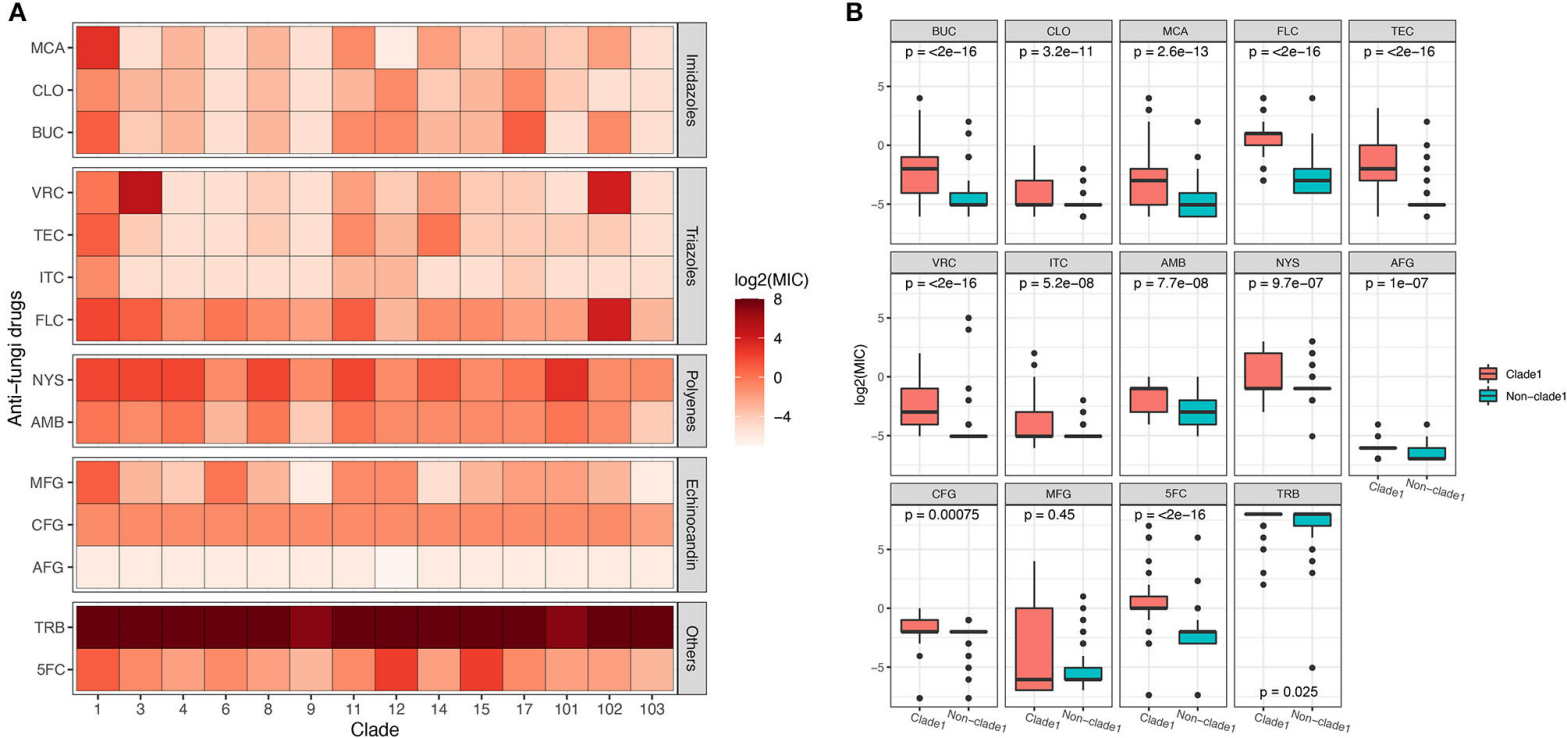
The susceptibility of *C. albicans* isolates in MLST clade 1 to antifungal drugs was significantly lower than that of non-clade 1 isolates. The MIC90 of each clade for 14 different antifungal drugs is presented **(A)**, revealing that the drug resistance of isolates from clade 1 was higher than that of other isolates. The Wilcoxon test was performed based on the MICs of each strain between clade 1 and non-clade 1 **(B)**. The resistance to all the antifungal drugs of the strains in clade 1 was significantly higher than that of non-clade 1 strains, except for resistance to MFG.

The MICs of 14 different drugs were determined for each *C. albicans* isolate (Supplementary Table 3). The MIC90 for each MLST clade was calculated and is shown in Figure 3. In general, all of the strains were sensitive to AFG but resistant to TRB. Although some non-clade 1 strains presented the highest resistance to several antifungal drugs, such as the clade 3 resistance to VRC and clades 12 and 15 resistance to 5FC, MLST clade 1 isolates showed the broadest spectrum of antifungal drug resistance. In addition, two of the three new clades discovered in this study, clades 101 and 102, showed the highest tolerance to NYS and FLC, respectively. This result indicates that the *C. albicans* in these two new clades not only had distinct genotypes but also exhibited different phenotypes. Furthermore, the difference in antifungal susceptibility was calculated between MLST clade 1 and non-clade 1 isolates, and the MIC values of clade 1 for all of the examined drugs were significantly higher than those of non-clade 1 strains, except that for MFG (Figure 3B). In addition, the MIC values of *C. albicans* strains in each MLST clade for the antifungal drugs are presented in Supplementary Figure 2 and Supplementary Table 3, and the statistical analysis based on Wilcoxon tests between clades also showed significantly different resistance abilities in clade 1. Interestingly, the new clade 102 manifested significantly higher VRC and FLC resistance than the other clades.

**Figure 4:**
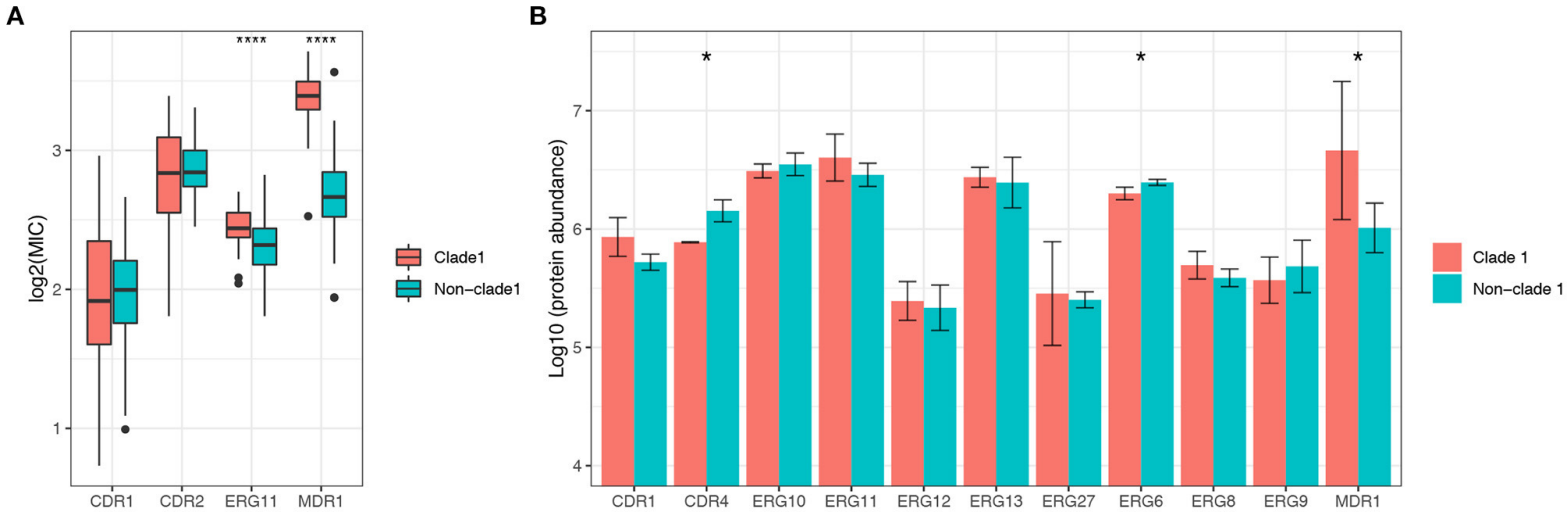
The expression level of the multidrug resistance gene MDR1 of *C. albicans* in MLST clade 1 isolates was significantly higher than that in non-clade 1 isolates. The mRNA expression levels of ERG11, CDR1, CDR2, and MDR1 were determined in 306 isolates **(A)**, while ERG11 and MDR1 exhibited significantly higher expressions in clade 1 than in other clades (***p < 0.0001). Consistently, the proteomics results **(B)** also showed that the expression of MDR1 protein was significantly higher in clade 1 (*p < 0.05). The CDR4 and ERG6 proteins also presented significant differences in expression between clade 1 and non-clade 1 isolates.

The mRNA expression of antifungal resistance genes was determined in this study to explore the mechanisms of the molecular resistance of *C. albicans* in different genotypes, as well as the expression of the proteins (Supplementary Figures 3A,B). Overall, the mRNA expression levels of resistance genes varied among different clades. The expression levels of the MDR1 and ERG11 genes were significantly higher (*p* < 0.0001) in MLST clade 1 strains than in non-clade 1 strains, while genes belonging to the CDR family showed no significant difference between clade 1 and other clades (Figure 4A). Proteins encoded by the MDR, ERG, and CDR gene families were also annotated by proteomic analysis, while only the protein amount of MDR1 was significantly different between MLST clade 1 strains and non-clade 1 strains, which is consistent with the corresponding transcriptional results (Figure 4B).

### 3.4. Higher Virulence Characteristics Exhibited by MLST Clade 1 Isolates

**Figure 5:**
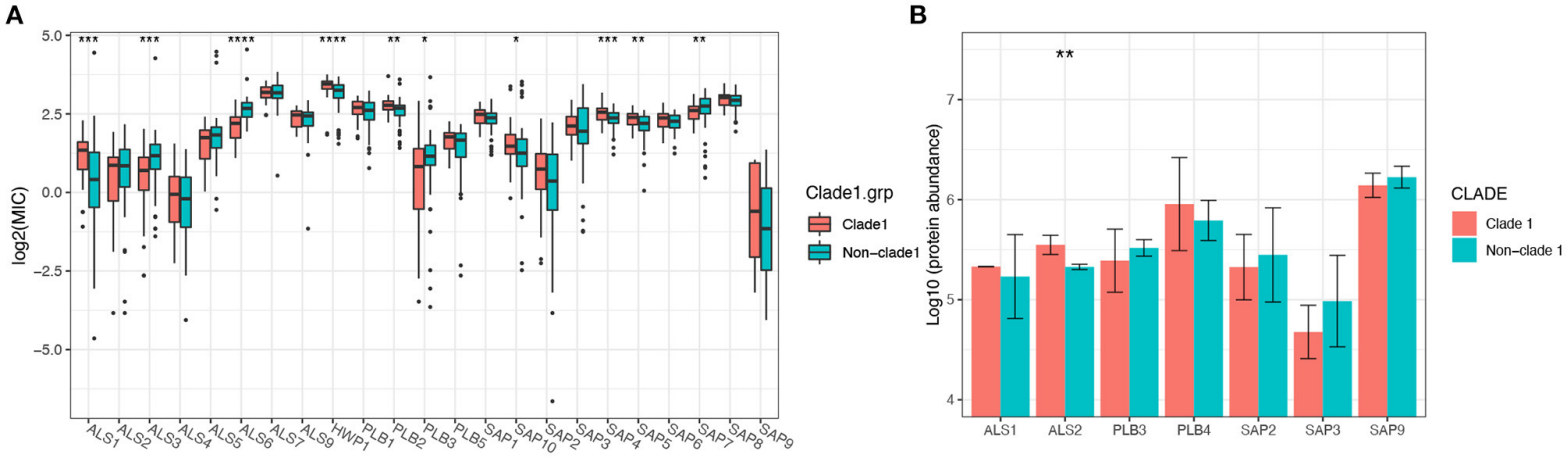
The mRNA expression levels of the virulence genes of *C. albicans* isolates varied significantly between MLST clade 1 and non-clade 1, while only ALS2 presented a significantly higher protein expression level. In total, 10 out of 23 genes from four virulence families, including SAP, HWP, PLB, and ALS, showed significantly different mRNA expression levels between clade 1 and non-clade 1 **(A)**. However, only seven proteins from these four virulence gene families were detected by proteomic analysis **(B)**, and ALS2 showed a significant difference between clade 1 and non-clade 1. *p < 0.05. **p < 0.01. ***p < 0.001. ****p < 0.0001.

The expression levels of virulence-related genes from the secretory aspartyl proteinase (SAP), hyphal wall protein (HWP), PLB, and ALS families were determined, and the results indicated that the virulence transcriptional pattern varied among different clades (Supplementary Figures 3C,D), as 10 out of 23 genes showed significantly different mRNA expression levels between clade 1 and non-clade 1 (Figure 5A). However, only seven proteins from these four virulence gene families were detected by proteomic analysis (Figure 5B), and the statistical analysis results were different. The ALS1 protein was expressed at non-significantly higher levels in clade 1, but its mRNA expression was significantly higher in clade 1 strains. The ALS2 protein was expressed at significantly higher levels in clade 1, but no significant difference was shown in mRNA expression. Moreover, all of the tested *C. albicans* strains were positive for phospholipase, esterase and hemolysis enzyme production, whereas there were no statistically significant differences in extracellular enzymatic activity among the different genotypes of *C. albicans* (Supplementary Figure 4 and Supplementary Table 4).

### 3.5. Differences in the Proteomic Expression Profile Between Clade 1 and Non-clade 1 *C. albicans* Isolates

**Figure 6:**
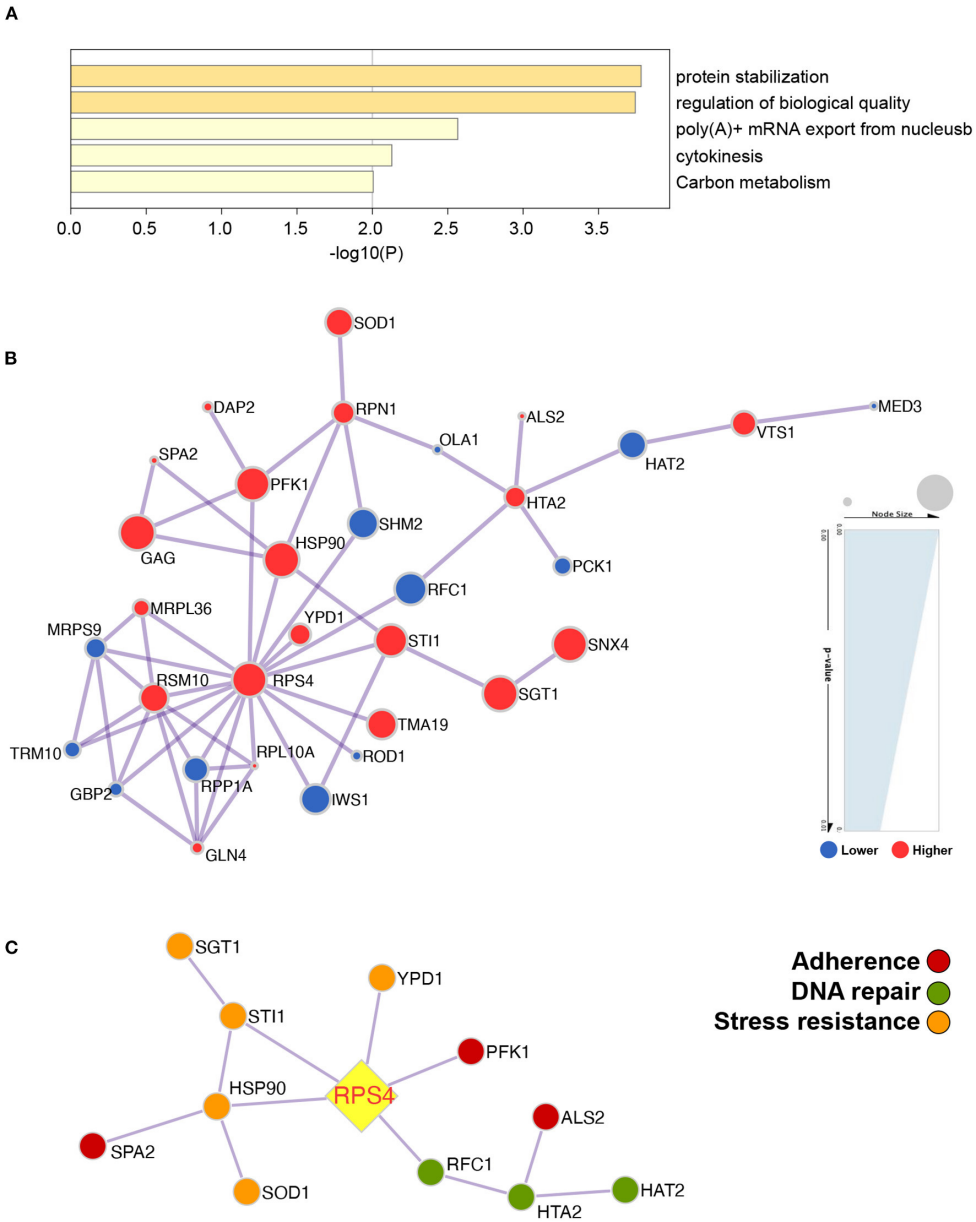
The protein profile demonstrated that the metabolic pathways were significantly different between *C. albicans* clade 1 isolates and non-clade 1 isolates and that RPS4 was the key gene linked to the proteins influencing the pathogenicity of *C. albicans*. The differentially expressed proteins were enriched in the pathways of protein stabilization, regulation of biological quality, poly(A)+ mRNA export from the nucleus, cytokinesis, and carbon metabolism **(A)**. The network of the proteins that were expressed significantly differently between clade 1 and non-clade 1 isolates was analyzed **(B)**, and RPS4 was the key protein associated with the stress resistance (HSP90, SGT1, SOD1, STI1, and YPD1), adherence (ALS2, PFK1, and SPA2), and DNA repair (HAT2, RFC1, and HTA2) of *C. albicans* **(C)**.

In total, 1,884 proteins were identified by proteomic analysis, and 1,677 proteins were subcategorized based on the GO classification biological process (23), cellular component (10), and molecular function (12). After statistical analysis, 41 proteins were found to be significantly differentially expressed between *C. albicans* MLST clade 1 isolates and non-clade 1 isolates (fold change > 2, *p* < 0.01, false discovery rate (FDR) < 0.01), among which 21 proteins were expressed at significantly higher levels in clade 1 isolates. The differentially expressed proteins were significantly enriched in five metabolic pathways, protein stabilization, the regulation of biological quality, poly(A)+ mRNA export from the nucleus, cytokinesis, and carbon metabolism (Figure 6A), which are pathways that contribute to tolerance, adaptation, and proliferation. The relationship between the differentially expressed proteins was analyzed, and the protein encoded by the RPS4 gene was discovered to be a central regulator that correlated with the proteins involved in the properties that influenced the resistance and virulence of the strain, such as stress resistance (HSP90, SGT1, SOD1, STI1, and YPD1), adherence (ALS2, PFK1, and SPA2), and DNA repair (HAT2, RFC1, and HTA2) (Figures 6B,C).

## 4. Discussion

The MLST used in this study is based on nucleotide sequence analysis of the internal regions of multiple independent housekeeping genes and has proven to be a useful method for epidemiological investigation of clinical isolates of *C. albicans*. MLST studies have been conducted on *C. albicans* isolates from the digestive tract, blood infections, oral leukoplakia, and vaginal secretions (33–37). In addition, MLST has been applied widely to study nosocomial infection, which is important for the prevention and control of infection (38, 39). Therefore, fine genotyping for clinically pathogenic *C. albicans* has important implications for the epidemiology of candidal infections, especially for infections with high incidence, such as VVC. The genotype of *C. albicans* isolates collected from the vaginal samples of Chinese VVC patients and asymptomatic volunteers was explored in a previous study, and the results showed that 71.6% of the VVC isolates were classified in MLST clade 1, while a significantly lower proportion (40.6%) of the vaginal isolates from asymptomatic volunteers were classified in clade 1 (5). In our current study, MLST clade 1 was also the most common group in isolates from patients with VVC, which indicated that additional attention should be given to the difference between *C. albicans* MLST clade 1 and non-clade 1 strains. In contrast to previous studies, the clinical characteristics of VVC patients were examined in this study. We found that clade 1 isolates accounted for the highest percentage of the RVVC and treatment failure cases, which demonstrated that VVC caused by invasive *C. albicans* belonging to clade 1 was more difficult to cure and more likely to relapse. This finding suggests that novel treatment methods need to be proposed to prevent VVC recurrence, such as vaginal microbiota regulation or immunotherapies.

The *C. albicans* strains of VVC treatment failure cases were also evaluated in this study. The MLST genotypes of the strains isolated from samples from recurrent cases were the same as those of the original infecting strains, which were mostly from MLST clade 1. Recurrence could be caused by uneradicated candida or repeated infection of candida from the same living and working environment due to its conserved genetic signatures and strong infection ability. Moorhouse et al. performed MLST on 42 *C. albicans* strains from six patients with chronic cutaneous candida disease at multiple body sites across multiple time points over 17 months and found that the genotype of strains isolated from each individual patient was stable (40). It has also been confirmed that *C. albicans* can stay in one location for a long time and occasionally show microvariation (41). Notably, this genotype characteristic was more likely to occur in MLST clade 1 strains, which indicated that the strains may have higher resistance and growth abilities with stable inheritance.

The development of antifungal resistance in *C. albicans* is one of the crucial reasons for treatment failure and recurrence. Hence, it is necessary for clinicians to understand the local distribution of *C. albicans* susceptibility to antifungal drugs prior to treatment initiation. It has been reported that the decreasing cure rate of VVC was closely related to the increasing use of azoles (42, 43), and the susceptibility profiles have varied in different cohorts (43, 44). Moreover, the genotype of *C. albicans* was also associated with susceptibility. For instance, resistance to FLC and TRB is associated with MTL homozygosity and MLST clade 1 (45). In this study, *C. albicans* MLST clade 1 strains had a distinct and broader spectrum of resistance to antifungal drugs, with MIC90 values of all drugs that were significantly higher than those of non-clade 1 strains, except for MFG. This result suggested that MFG may be a better choice for the treatment of clade 1 strain infection. Furthermore, taking the molecular mechanisms of antifungal resistance into consideration, the ABC transporter CDR1/CDR2 and multidrug transporter MDR1, which are responsible for reducing intracellular drug levels via efflux pump activity, and one of the drug targets, ergosterol synthase ERG11, were selected to evaluate their mRNA expression levels in strains with different genotypes and susceptibilities, as well as the levels of proteins from the same families. MDR1 was found to be significantly more highly expressed in clade 1 strains than in non-clade 1 strains at both the transcriptional and translational levels. Therefore, the multidrug transporter could be the key mechanism for the resistance of MLST clade 1 strains.

The virulence factors of *C. albicans* contribute to VVC clinical characteristics by regulating adhesion, invasion and damage to host epithelial cells. Many proteins, including ALS proteins, phospholipase B (PLB), SAPs, and HWPs, mediate pathogen virulence (46), as well as some extracellular enzyme production (47). Our study showed that the transcriptional levels of SAP4, SAP5, SAP7, and SAP10 were significantly different in MLST clade 1 strains. Among them, SAP4 and SAP5 are involved in adherence, mucosal damage and the immune response of the host, while SAP10 maintains cell wall integrity. Moreover, in *C. albicans* MLST clade 1 strains, ALS1, ALS3, and ALS6 presented significantly higher mRNA expression levels, and ALS2 presented significantly higher protein expression levels. Genes of the ALS family participate in the pathogenic process of *C. albicans* by adhering to the host (48), and HWP1 expression also appeared to be significantly different. Although PLB2 and PLB3, which are involved in phospholipase synthesis, were overexpressed in clade 1 strains, their production showed no significant difference among the genotypes of *C. albicans*.

To further understand the mechanisms that drive the higher resistance and virulence of *C. albicans* in MLST clade 1, proteomic analysis was applied in this study. Proteins involved in the protein stabilization pathway were determined to be enriched in clade 1 strains, which should contribute to strain resistance against stress and the maintenance of regular growth and virulence. Based on the interaction network analysis of the differentially expressed proteins, a key gene, RPS4, was found to be linked with the proteins influencing the resistance and virulence of *C. albicans*. RPS4 is a gene encoding a ribosomal protein responsible for cellular protein biosynthesis. However, little is known about how the ribosome operates to mediate microbial pathogenesis. Recently, a pre-rRNA processing factor was verified to be a regulator of fungal development and pathogenesis by mediating the production of reactive oxygen species (49). Moreover, the expression of RPS4 has more than a twofold difference during biofilm development in *C. albicans* (50). In the current study, RPS4 was associated with the expression of HSP90, SGT1, STI1, SOD1, and YPD1, which are all involved in stress resistance. HSP90 is a heat shock protein that mediates the drug resistance pathways of *C. albicans* via various signaling pathways by binding with different cochaperones, such as SGT1 and STI, as discovered in this study (51–53). SOD1 and YPD1 have been reported to protect *C. albicans* against oxidative stress and maintain the virulence of strains (54, 55). In addition, the ALS2, SPA2, and PFK1 proteins that influence the adherence of the strains have been linked with RPS4 directly or indirectly (56–58). Furthermore, DNA repair proteins (HAT2, RFC1, and HTA2) might also be regulated by RPS4 in MLST clade 1 *C. albicans* and affect the growth stability of the strains.

## 5. Conclusion

The MLST of *C. albicans* is closely associated with clinical phenotypes. Among the 306 isolated strains from VVC patients, MLST clade 1 was the most common group and accounted for the highest proportion of RVVC and treatment failure cases. In addition, significantly higher antifungal resistance was shown in MLST clade 1 isolates. The mRNA of the genes and encoded proteins related to drug resistance and virulence were also expressed at significantly higher levels in clade 1 strains, indicating a higher pathogenicity, which corresponded with the clinical phenotype. The proteomic analysis indicated that these properties of *C. albicans* MLST clade 1 were associated with the protein stabilization pathway. Furthermore, RSP4 is a central regulator that influences the pathogenicity of clade 1 strains by linking to genes involved in stress resistance, adherence and DNA repair.

## Data Availability Statement

The datasets presented in this study can be found in online repositories. The names of the repository/repositories and accession number(s) can be found at: https://www.iprox.cn/page/project.html?id=IPX0003855000.

## Ethics Statement

The studies involving human participants were reviewed and approved by Medical Review Board of Peking University Shenzhen Hospital. The patients/participants provided their written informed consent to participate in this study.

## Author Contributions

SF and XZ: conceptualization and investigation. YZhu, CF, YShi, XiaL, and XZ: data curation. YZhu, CF, and XZ: formal analysis. SF: funding acquisition and supervision. YZhu, YSha, YL, LH, XinL and CL: methodology. YZhu and SF: project administration. YZhu, YSha, YL, LH, and SF: resources. YZhu and CF: software. YShi, XiaL, YL, and YZha: validation. CF: visualization. YZhu and XZ: writing—original draft. CF and XZ: writing—review and editing.

## Funding

This research was funded by the Science and Technology Planning Project of Shenzhen Municipality (Grant Numbers JCYJ20180228162311024 and JCYJ20200109140614667) and the National Natural Science Foundation of China (Grant Number 82171676).

## Conflict of Interest

CF was employed by BGI-Shenzhen. The remaining authors declare that the research was conducted in the absence of any commercial or financial relationships that could be construed as a potential conflict of interest.

## Publisher's Note

All claims expressed in this article are solely those of the authors and do not necessarily represent those of their affiliated organizations, or those of the publisher, the editors and the reviewers. Any product that may be evaluated in this article, or claim that may be made by its manufacturer, is not guaranteed or endorsed by the publisher.

## Abbreviations

VVC: vulvovaginal candidiasis
RVVC: recurrent vulvovaginal candidiasis
MLST: multilocus sequence typing
MTL: mating type locus
DST: diploid sequence type
UPGMA: unweighted-pair group method using average linkages
AAT1a: aspartate aminotransferase
ACC1: acetyl-coenzyme A carboxylase
ADP1: ATP-dependent permease
MPIb: mannose phosphate isomerase
SYA1: alanyl-RNA synthetase
VPS13: vacuolar protein sorting protein
ZWF1b: glucose-6-phosphate dehydrogenase
CDR: ATP-binding cassette multidrug transporter
MDR: multidrug resistance gene
ERG: ergosterol biosynthesis gene
ALS: agglutinin-like sequence
HWP: hyphal wall protein
SAP: secreted aspartyl protease
PLB: phospholipase B
ITC: itraconazole
MCA: miconazole
CLO: clotrimazole
VRC: voriconazole
TEC: terconazole
BUC: butoconazole
AMB: amphotericin B
TRB: terbinafine
CFG: caspofungin
MFG: micafungin
AFG: anidulafungin
FLC: fluconazole
NYS: nystatin
5FC: 5-fluorocytosine
MIC: minimum inhibitory concentration.
