# Supplementary material for: Candida albicans Multilocus Sequence Typing Clade I Contributes to the Clinical Phenotype of Vulvovaginal Candidiasis Patients
Source: Front Med (Lausanne). 2022 Apr 1;9:837536. doi: 10.3389/fmed.2022.837536 (PMC9010739; doi:10.3389/fmed.2022.837536)
Supplement: Supplementary file 1 [file Data_Sheet_1.pdf]

# Supplementary Material

## 1 SUPPLEMENTARY TABLES AND FIGURES

### 1.1 Figures

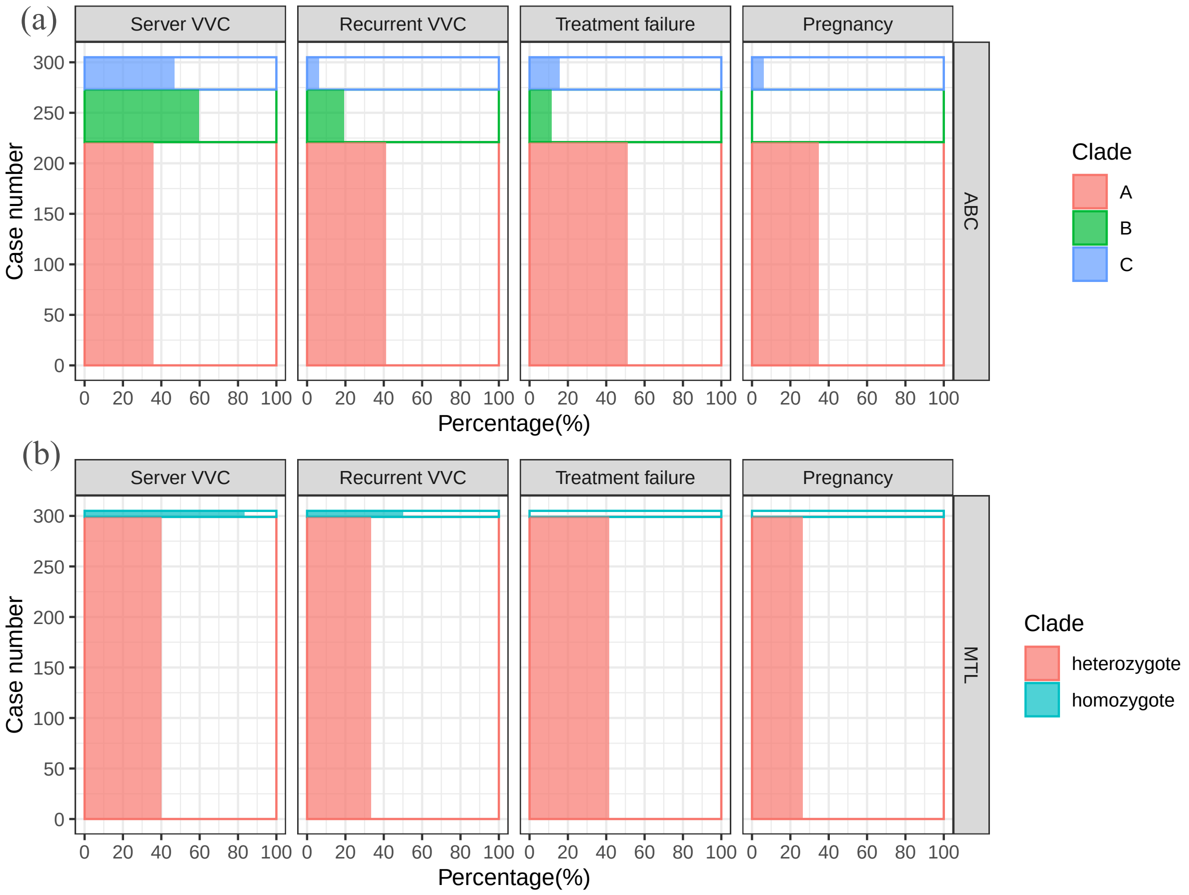


**Figure S1. Clinical characteristics of VVC caused by *C. albicans* with different ABC genotypes and MTL ho-mozygosis.**

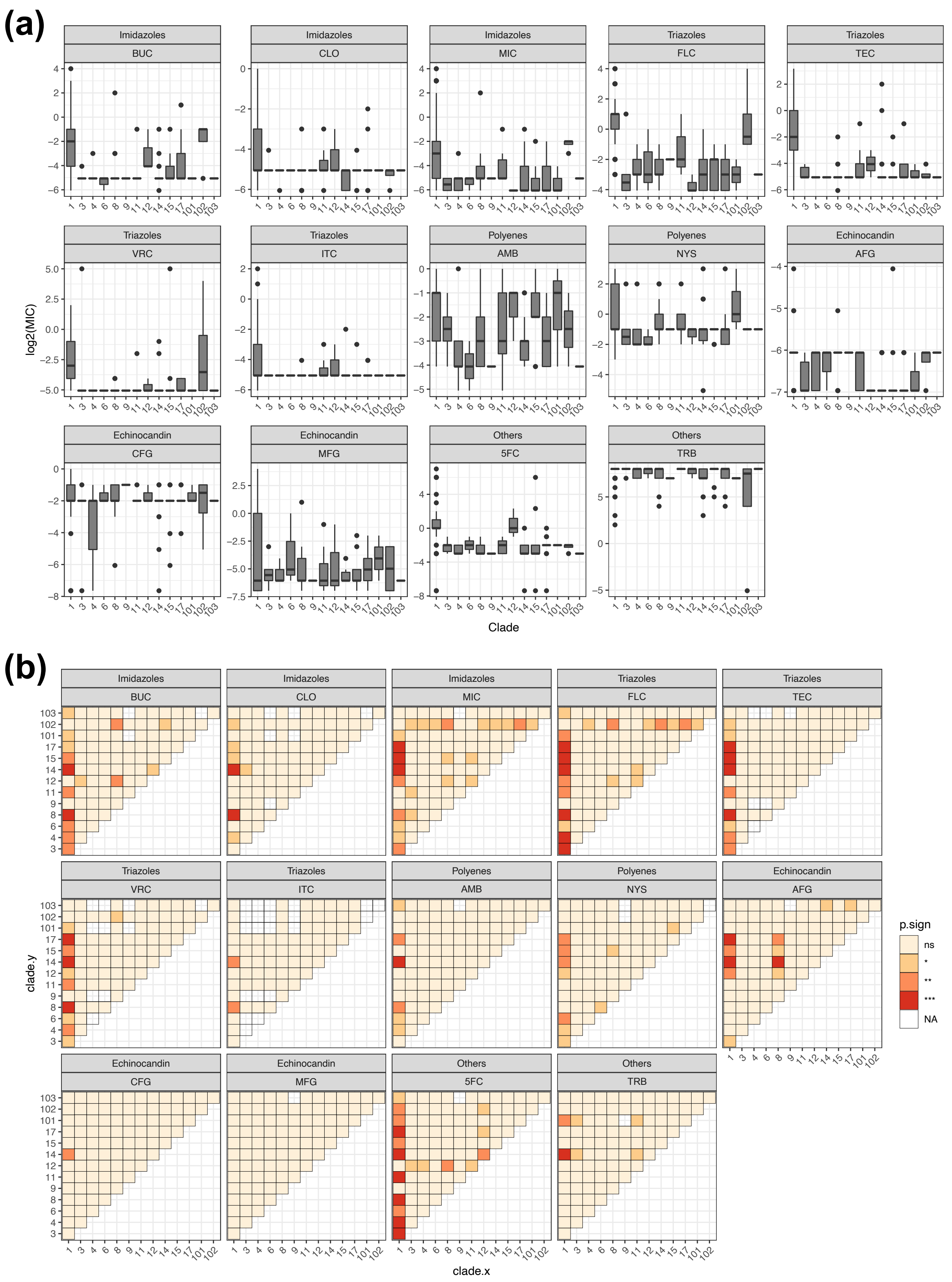


**Figure S2. The MIC90 of C. albicans strains in each MLST clade for the antifungal drugs (a) and the *p-value* generated by a Wilcoxon test (b) are presented.** No significant difference is denoted by ns. *p-value* $< 0.05$ is denoted by *. *p-value* $< 0.01$ is denoted by **. *p-value* $< 0.001$ is denoted by ***. No data shown is denoted by NA. All the correlated colors shown in the legend are filled in the heatmap.

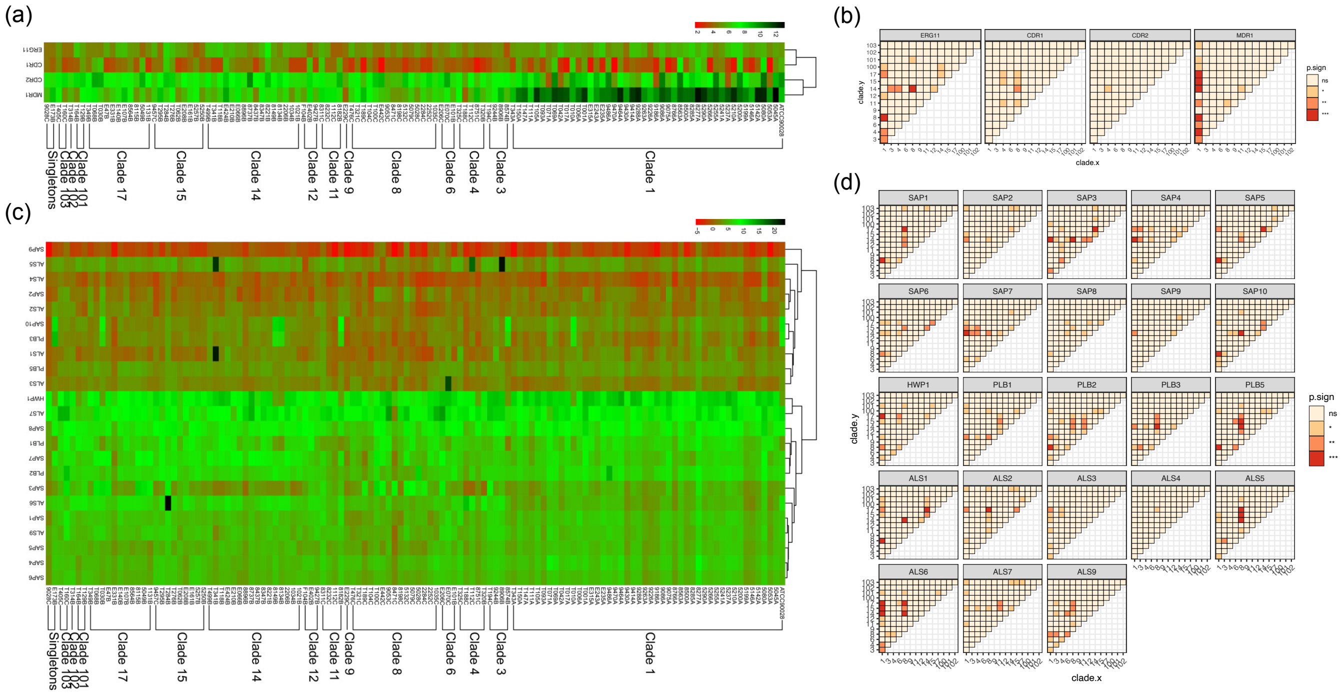


**Figure S3. The mRNA expression level of each *C. albicans* strain (a) and the *p-value* between each MLST clade generated by a Wilcoxon test (b) are presented.** No significant difference is denoted by ns. *p-value* $< 0.05$ is denoted by *. *p-value* $< 0.01$ is denoted by **. *p-value* $< 0.001$ is denoted by ***. No data shown is denoted by NA. All the correlated colors shown in the legend are filled in the heatmap.

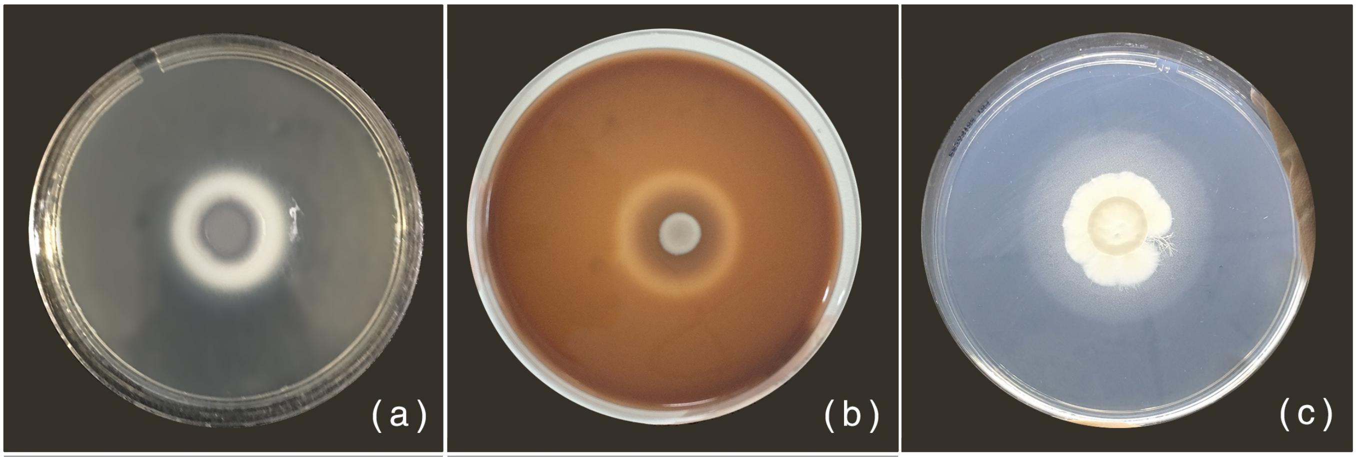


**Figure S4. The test results of *C. albicans* strain were positive for phospholipase (a), hemolysis enzyme (b) and esterase (c) production.**

## 1.2 Tables

Table S1: The MLST, ABC genotype, and MTL homozygosis of the *C. albicans* isolates. * stands for a new MLST clade identified in this study.

| Begin of Table S1 | | | | | | | | | | | |
|---|---|---|---|---|---|---|---|---|---|---|---|
| Strain | AAT1a | ACC1 | ADP1 | MPIb | SYA1 | VPS13 | ZWF1b | Eburst cluster | MLSTClade | ABC Type | MTL type |
| T499B | 3 | 3 | 21 | 160 | 53 | 109 | 13 | 15 | 14 | B | het |
| T476C | 1 | 3 | 4 | 3 | 7 | 73 | 112 | 21 | 8 | C | het |

| Continuation of Table S1 | | | | | | | | | | | |
|---|---|---|---|---|---|---|---|---|---|---|---|
| Strain | AAT1a | ACC1 | ADP1 | MPIb | SYA1 | VPS13 | ZWF1b | Eburst cluster | MLSTClade | ABC Type | MTL type |
| T405C | 13 | 3 | 4 | 6 | 34 | 20 | 18 | 41 | 103 | C | het |
| T349B | 59 | 7 | 21 | 2 | 81 | 108 | 90 | 32 | 17 | B | het |
| T343A | 2 | 5 | 5 | 9 | 2 | 6 | 5 | 1 | 1 | A | het |
| T341B | 3 | 3 | 21 | 50 | 53 | 109 | 13 | 15 | 14 | B | het |
| T325C | 74 | 14 | 6 | 4 | 30 | 112 | 8 | 125 | 4 | C | het |
| T321C | 1 | 3 | 6 | 3 | 7 | 73 | 112 | 21 | 8 | C | het |
| T320B | 11 | 26 | 6 | 4 | 34 | 60 | 119 | 12 | 4 | B | het |
| T314B | 14 | 46 | 21 | 2 | 26 | 287 | 272 | S | 102* | B | het |
| T295B | 13 | 3 | 6 | 108 | 7 | 105 | 12 | 18 | 15 | B | het |
| T284B | 13 | 80 | 6 | 108 | 7 | 105 | 12 | 18 | 15 | B | het |
| T276B | 13 | 80 | 6 | 94 | 7 | 105 | 12 | 18 | 15 | B | het |
| T194C | 109 | 68 | 15 | 34 | 61 | 291 | 209 | 137 | 3 | C | het |
| T189C | 24 | 7 | 6 | 9 | 34 | 230 | 15 | S | 8 | C | het |
| T188C | 11 | 26 | 6 | 4 | 34 | 60 | 119 | 12 | 4 | C | het |
| T164B | 21 | 7 | 6 | 2 | 45 | 51 | 47 | S | 101* | B | het |
| T160C | 4 | 26 | 6 | 18 | 24 | 27 | 112 | 73 | 103* | C | het |
| T150A | 2 | 5 | 5 | 9 | 2 | 24 | 5 | 1 | 1 | A | het |
| T147A | 2 | 5 | 5 | 9 | 2 | 6 | 5 | 1 | 1 | A | het |
| T129B | 80 | 18 | 6 | 2 | 30 | 136 | 271 | S | 101* | B | het |
| T118B | 6 | 3 | 6 | 4 | 53 | 109 | 13 | 15 | 14 | B | het |
| T112C | 11 | 26 | 6 | 4 | 34 | 60 | 119 | 12 | 4 | C | het |
| T111A | 2 | 2 | 5 | 9 | 2 | 6 | 5 | 1 | 1 | A | het |
| T105A | 2 | 5 | 5 | 9 | 2 | 6 | 5 | 1 | 1 | A | het |
| T104C | 1 | 3 | 4 | 3 | 7 | 73 | 112 | 21 | 8 | C | het |
| T100C | 55 | 8 | 6 | 2 | 34 | 94 | 15 | 138 | 8 | C | het |
| T093A | 2 | 3 | 90 | 9 | 2 | 6 | 5 | 1 | 1 | A | het |
| T071A | 2 | 3 | 90 | 9 | 2 | 6 | 5 | 1 | 1 | A | het |
| T069A | 2 | 5 | 5 | 9 | 2 | 6 | 5 | 1 | 1 | A | het |
| T068B | 59 | 110 | 10 | 2 | 80 | 108 | 47 | S | 17 | B | het |
| T062B | 47 | 14 | 99 | 50 | 6 | 30 | 12 | 77 | 15 | B | het |
| T042A | 2 | 5 | 90 | 9 | 2 | 6 | 5 | 1 | 1 | A | het |
| T030B | 59 | 3 | 10 | 2 | 80 | 108 | 15 | 13 | 17 | B | het |
| T017A | 2 | 3 | 90 | 9 | 2 | 6 | 5 | 1 | 1 | A | het |
| T010A | 2 | 5 | 5 | 9 | 2 | 6 | 5 | 1 | 1 | A | het |
| T006A | 2 | 5 | 5 | 9 | 2 | 6 | 5 | 1 | 1 | A | het |
| T001A | 2 | 3 | 90 | 9 | 2 | 6 | 5 | 1 | 1 | A | het |
| P152-2 | 60 | 10 | 21 | 1 | 34 | 126 | 15 | 6 | 11 | C | het |
| P152 | 60 | 10 | 21 | 1 | 34 | 126 | 15 | 6 | 11 | C | het |
| P145-2 | 14 | 3 | 6 | 4 | 7 | 13 | 8 | 2 | 4 | A | het |
| P145 | 14 | 3 | 6 | 4 | 7 | 13 | 8 | 2 | 4 | A | het |
| P144-2 | 2 | 5 | 5 | 9 | 2 | 6 | 5 | 1 | 1 | A | het |
| P144 | 2 | 5 | 5 | 9 | 2 | 6 | 5 | 1 | 1 | A | het |
| P139-2 | 8 | 5 | 5 | 9 | 2 | 6 | 5 | 1 | 1 | A | het |
| P139 | 2 | 5 | 5 | 9 | 2 | 6 | 5 | 1 | 1 | A | het |
| P137-2 | 2 | 5 | 5 | 9 | 2 | 6 | 5 | 1 | 1 | A | het |
| P137 | 2 | 5 | 5 | 9 | 2 | 6 | 5 | 1 | 1 | A | het |
| P135-2 | 2 | 5 | 5 | 9 | 2 | 6 | 5 | 1 | 1 | A | het |

| Continuation of Table S1 | | | | | | | | | | | |
|---|---|---|---|---|---|---|---|---|---|---|---|
| Strain | AAT1a | ACC1 | ADP1 | MPIb | SYA1 | VPS13 | ZWF1b | Eburst cluster | MLSTClade | ABC Type | MTL type |
| P135 | 2 | 5 | 5 | 9 | 2 | 6 | 5 | 1 | 1 | A | het |
| P134-3 | 1 | 3 | 4 | 3 | 7 | 73 | 112 | 21 | 8 | A | het |
| P134 | 1 | 3 | 4 | 3 | 7 | 73 | 112 | 21 | 8 | A | het |
| P133-2 | 2 | 5 | 5 | 9 | 2 | 6 | 5 | 1 | 1 | A | het |
| P133 | 2 | 5 | 5 | 9 | 2 | 6 | 5 | 1 | 1 | A | het |
| P128-5 | 2 | 5 | 5 | 9 | 2 | 6 | 5 | 1 | 1 | A | het |
| P128-3 | 2 | 5 | 5 | 9 | 2 | 6 | 5 | 1 | 1 | A | het |
| P128-2 | 2 | 5 | 5 | 9 | 2 | 6 | 5 | 1 | 1 | A | het |
| P128 | 2 | 5 | 5 | 9 | 2 | 6 | 5 | 1 | 1 | A | het |
| P122-4 | 2 | 78 | 2 | 9 | 2 | 24 | 5 | 1 | 1 | A | het |
| P122 | 2 | 78 | 2 | 9 | 2 | 24 | 5 | 1 | 1 | A | het |
| P121-2 | 2 | 5 | 5 | 9 | 2 | 6 | 5 | 1 | 1 | A | het |
| P121 | 2 | 5 | 5 | 9 | 2 | 6 | 5 | 1 | 1 | A | het |
| P107-2 | 2 | 3 | 90 | 9 | 2 | 6 | 5 | 1 | 1 | A | het |
| P107 | 2 | 3 | 90 | 9 | 2 | 6 | 5 | 1 | 1 | A | het |
| P085-3 | 2 | 78 | 5 | 9 | 2 | 6 | 5 | 1 | 1 | A | het |
| P085-2 | 2 | 78 | 5 | 9 | 2 | 6 | 5 | 1 | 1 | A | het |
| P085 | 2 | 78 | 5 | 9 | 2 | 6 | 5 | 1 | 1 | A | het |
| P079-2 | 2 | 5 | 5 | 9 | 2 | 6 | 5 | 1 | 1 | A | het |
| P079 | 2 | 5 | 5 | 9 | 2 | 6 | 5 | 1 | 1 | A | het |
| P074-3 | 2 | 5 | 5 | 9 | 2 | 6 | 5 | 1 | 1 | A | het |
| P074-2 | 2 | 5 | 5 | 9 | 2 | 6 | 5 | 1 | 1 | A | het |
| P074 | 2 | 5 | 5 | 9 | 2 | 6 | 5 | 1 | 1 | A | het |
| P072-3 | 2 | 5 | 5 | 9 | 2 | 6 | 5 | 1 | 1 | A | het |
| P072 | 2 | 5 | 5 | 9 | 2 | 6 | 5 | 1 | 1 | A | het |
| P070-3 | 2 | 78 | 5 | 9 | 2 | 6 | 5 | 1 | 1 | A | het |
| P070-2 | 2 | 78 | 5 | 9 | 2 | 6 | 5 | 1 | 1 | A | het |
| P070 | 2 | 78 | 5 | 9 | 2 | 6 | 5 | 1 | 1 | A | het |
| P067-2 | 8 | 5 | 5 | 9 | 2 | 6 | 5 | 1 | 1 | A | het |
| P067 | 8 | 5 | 5 | 9 | 2 | 6 | 5 | 1 | 1 | A | het |
| P065-3 | 2 | 5 | 5 | 9 | 2 | 6 | 5 | 1 | 1 | A | het |
| P065-2 | 2 | 5 | 5 | 9 | 2 | 6 | 5 | 1 | 1 | A | het |
| P065 | 2 | 5 | 5 | 9 | 2 | 6 | 5 | 1 | 1 | A | het |
| P060-2 | 5 | 10 | 37 | 4 | 34 | 105 | 22 | 133 | 3 | A | het |
| P060 | 5 | 10 | 37 | 4 | 34 | 105 | 22 | 133 | 3 | A | het |
| P054-2 | 2 | 5 | 5 | 9 | 2 | 6 | 5 | 1 | 1 | A | het |
| P054 | 2 | 5 | 5 | 9 | 2 | 6 | 5 | 1 | 1 | A | het |
| P050-3 | 2 | 3 | 90 | 9 | 2 | 6 | 5 | 1 | 1 | A | het |
| P050-2 | 2 | 3 | 90 | 9 | 2 | 6 | 5 | 1 | 1 | A | het |
| P050 | 2 | 3 | 90 | 9 | 2 | 6 | 5 | 1 | 1 | A | het |
| P049-2 | 2 | 5 | 5 | 9 | 2 | 6 | 5 | 1 | 1 | A | het |
| P049 | 2 | 5 | 5 | 9 | 2 | 6 | 5 | 1 | 1 | A | het |
| P047-3 | 2 | 5 | 5 | 9 | 2 | 24 | 5 | 1 | 1 | A | het |
| P047-2 | 2 | 5 | 5 | 9 | 2 | 24 | 5 | 1 | 1 | A | het |
| P047 | 2 | 5 | 5 | 9 | 2 | 24 | 5 | 1 | 1 | A | het |
| P044-9 | 2 | 5 | 5 | 9 | 2 | 6 | 5 | 1 | 1 | A | het |
| P044-7 | 2 | 5 | 5 | 9 | 2 | 6 | 5 | 1 | 1 | A | het |

| Continuation of Table S1 | | | | | | | | | | | |
|---|---|---|---|---|---|---|---|---|---|---|---|
| Strain | AAT1a | ACC1 | ADP1 | MPIb | SYA1 | VPS13 | ZWF1b | Eburst cluster | MLSTClade | ABC Type | MTL type |
| P044-6 | 2 | 5 | 5 | 9 | 2 | 6 | 5 | 1 | 1 | A | het |
| P044-5 | 2 | 5 | 5 | 9 | 2 | 6 | 5 | 1 | 1 | A | het |
| P044-4 | 2 | 5 | 5 | 9 | 2 | 6 | 5 | 1 | 1 | A | het |
| P044-3 | 2 | 5 | 5 | 9 | 2 | 6 | 5 | 1 | 1 | A | het |
| P044-2 | 2 | 3 | 5 | 9 | 2 | 6 | 5 | 1 | 1 | A | het |
| P044 | 2 | 5 | 5 | 9 | 2 | 6 | 5 | 1 | 1 | A | het |
| P040-2 | 2 | 5 | 90 | 9 | 2 | 6 | 5 | 1 | 1 | A | het |
| P040 | 2 | 3 | 90 | 9 | 2 | 6 | 5 | 1 | 1 | A | het |
| P028-2 | 2 | 5 | 5 | 9 | 2 | 6 | 5 | 1 | 1 | A | het |
| P028 | 2 | 5 | 5 | 9 | 2 | 6 | 5 | 1 | 1 | A | het |
| P019-3 | 13 | 26 | 5 | 3 | 93 | 53 | 12 | 29 | 1 | A | het |
| P019-2 | 13 | 26 | 5 | 3 | 93 | 53 | 12 | 29 | 1 | A | het |
| P019 | 13 | 26 | 5 | 3 | 93 | 53 | 12 | 29 | 1 | A | het |
| P015-2 | 2 | 5 | 5 | 9 | 2 | 6 | 5 | 1 | 1 | A | het |
| P015 | 2 | 5 | 5 | 9 | 2 | 6 | 5 | 1 | 1 | A | het |
| P014-3 | 2 | 5 | 5 | 9 | 2 | 6 | 5 | 1 | 1 | A | het |
| P014-2 | 2 | 5 | 5 | 9 | 2 | 6 | 5 | 1 | 1 | A | het |
| P014 | 2 | 5 | 5 | 9 | 2 | 6 | 5 | 1 | 1 | A | het |
| P005-4 | 55 | 14 | 4 | 3 | 6 | 45 | 15 | 5 | 8 | A | het |
| P005-3 | 55 | 14 | 4 | 3 | 6 | 45 | 15 | 5 | 8 | A | het |
| P005 | 55 | 14 | 4 | 3 | 6 | 45 | 15 | 5 | 8 | A | het |
| G237 | 2 | 3 | 90 | 9 | 2 | 6 | 5 | 1 | 1 | A | hom$\alpha$ |
| F104B | 4 | 17 | 21 | 19 | 30 | 83 | 22 | 7 | 12 | B | het |
| E47B | 59 | 5 | 21 | 2 | 80 | 108 | 15 | 13 | 17 | B | het |
| E465-4 | 2 | 3 | 90 | 9 | 2 | 6 | 5 | 1 | 1 | A | het |
| E465-3 | 2 | 3 | 90 | 9 | 2 | 6 | 5 | 1 | 1 | A | het |
| E449-5 | 2 | 2 | 5 | 9 | 2 | 6 | 5 | 1 | 1 | A | het |
| E449 | 2 | 2 | 5 | 9 | 2 | 6 | 5 | 1 | 1 | A | het |
| E442C | 24 | 7 | 6 | 9 | 34 | 230 | 15 | S | 8 | C | het |
| E428-7 | 2 | 5 | 5 | 9 | 2 | 6 | 5 | 1 | 1 | A | het |
| E428-6 | 2 | 5 | 5 | 9 | 2 | 6 | 5 | 1 | 1 | A | het |
| E424B | 6 | 3 | 21 | 50 | 27 | 109 | 13 | 15 | 14 | B | het |
| E424-4 | 3 | 3 | 21 | 50 | 27 | 109 | 13 | 15 | 14 | B | het |
| E424-3 | 6 | 3 | 21 | 50 | 27 | 109 | 13 | 15 | 14 | B | het |
| E423-5 | 2 | 3 | 90 | 9 | 2 | 6 | 5 | 1 | 1 | A | het |
| E423-4 | 2 | 3 | 90 | 9 | 2 | 6 | 5 | 1 | 1 | A | het |
| E423 | 2 | 3 | 90 | 9 | 2 | 6 | 5 | 1 | 1 | A | het |
| E402B | 4 | 17 | 21 | 19 | 27 | 83 | 22 | 7 | 12 | B | het |
| E393-5 | 2 | 3 | 90 | 9 | 2 | 6 | 5 | 1 | 1 | A | het |
| E393-4 | 2 | 5 | 5 | 9 | 2 | 6 | 5 | 1 | 1 | A | het |
| E393 | 2 | 3 | 90 | 9 | 2 | 6 | 5 | 1 | 1 | A | het |
| E336-5 | 2 | 5 | 5 | 9 | 2 | 6 | 5 | 1 | 1 | A | het |
| E336 | 2 | 5 | 5 | 9 | 2 | 6 | 5 | 1 | 1 | A | het |
| E331B | 59 | 7 | 21 | 2 | 81 | 20 | 90 | 32 | 17 | B | het |
| E315A | 2 | 5 | 5 | 2 | 2 | 6 | 5 | 1 | 1 | A | het |
| E309-3 | 2 | 5 | 5 | 9 | 2 | 6 | 5 | 1 | 1 | A | het |
| E309 | 2 | 3 | 90 | 9 | 2 | 6 | 5 | 1 | 1 | A | het |

| Continuation of Table S1 | | | | | | | | | | | |
|---|---|---|---|---|---|---|---|---|---|---|---|
| Strain | AAT1a | ACC1 | ADP1 | MPIb | SYA1 | VPS13 | ZWF1b | Eburst cluster | MLSTClade | ABC Type | MTL type |
| E306-3 | 2 | 3 | 90 | 9 | 2 | 6 | 5 | 1 | 1 | A | het |
| E306 | 2 | 3 | 90 | 9 | 2 | 6 | 5 | 1 | 1 | A | het |
| E286 | 2 | 3 | 90 | 9 | 2 | 6 | 5 | 1 | 1 | A | hom$\alpha$ |
| E277-5 | 2 | 3 | 5 | 9 | 2 | 6 | 5 | 1 | 1 | A | het |
| E277 | 2 | 3 | 5 | 9 | 2 | 6 | 5 | 1 | 1 | A | het |
| E275-4 | 2 | 5 | 5 | 9 | 2 | 6 | 5 | 1 | 1 | A | het |
| E275 | 2 | 5 | 5 | 9 | 2 | 6 | 5 | 1 | 1 | A | het |
| E243A | 2 | 5 | 5 | 2 | 2 | 6 | 5 | 1 | 1 | A | het |
| E235A | 2 | 5 | 90 | 9 | 2 | 6 | 5 | 1 | 1 | A | het |
| E229C | 33 | 3 | 3 | 3 | 3 | 39 | 95 | 8 | 9 | C | het |
| E221 | 2 | 3 | 90 | 9 | 2 | 6 | 5 | 1 | 1 | A | hom$\alpha$ |
| E211 | 21 | 17 | 21 | 19 | 27 | 83 | 22 | 7 | 12 | C | hom$\alpha$ |
| E210B | 6 | 3 | 21 | 50 | 53 | 109 | 13 | 15 | 14 | B | het |
| E208B | 13 | 80 | 6 | 108 | 74 | 105 | 12 | 18 | 15 | B | het |
| E206C | 29 | 7 | 6 | 3 | 43 | 230 | 12 | 43 | 6 | C | het |
| E196-10 | 2 | 5 | 5 | 9 | 2 | 6 | 5 | 1 | 1 | A | het |
| E196 | 2 | 5 | 5 | 9 | 2 | 6 | 5 | 1 | 1 | A | het |
| E191-8 | 2 | 5 | 5 | 9 | 2 | 6 | 5 | 1 | 1 | A | het |
| E191 | 2 | 5 | 5 | 9 | 2 | 6 | 5 | 1 | 1 | A | het |
| E188-10 | 2 | 78 | 5 | 9 | 2 | 6 | 5 | 1 | 1 | A | het |
| E188 | 2 | 78 | 5 | 9 | 2 | 6 | 5 | 1 | 1 | A | het |
| E173B | 47 | 35 | 4 | 21 | 74 | 118 | 105 | S | 100* | B | het |
| E165-9 | 10 | 14 | 21 | 2 | 158 | 12 | 253 | S | 102* | A | het |
| E165-8 | 10 | 14 | 21 | 2 | 158 | 12 | 253 | S | 102* | A | het |
| E165-11 | 10 | 14 | 21 | 2 | 158 | 12 | 253 | S | 102* | A | het |
| E165 | 2 | 5 | 5 | 9 | 2 | 6 | 5 | 1 | 1 | A | het |
| E161B | 13 | 80 | 6 | 108 | 2 | 105 | 12 | 18 | 15 | B | het |
| E140B | 59 | 7 | 21 | 2 | 81 | 108 | 90 | 32 | 17 | B | het |
| E107B | 59 | 7 | 21 | 2 | 81 | 108 | 90 | 32 | 17 | B | het |
| E101B | 21 | 26 | 14 | 18 | 72 | 102 | 12 | 16 | 6 | B | het |
| E070C | 21 | 26 | 25 | 9 | 38 | 65 | 12 | 104 | 6 | C | het |
| E066B | 3 | 3 | 21 | 50 | 27 | 109 | 13 | 15 | 14 | B | het |
| ATCC90028 | 2 | 3 | 5 | 9 | 2 | 6 | 12 | 1 | 1 | A | het |
| 9497-2 | 2 | 3 | 90 | 9 | 2 | 6 | 5 | 1 | 1 | A | het |
| 9497 | 2 | 3 | 90 | 9 | 2 | 6 | 5 | 1 | 1 | A | het |
| 9486A | 2 | 5 | 5 | 9 | 2 | 6 | 5 | 1 | 1 | A | het |
| 9470A | 2 | 3 | 90 | 9 | 2 | 6 | 5 | 1 | 1 | A | het |
| 9464A | 2 | 3 | 90 | 9 | 2 | 6 | 5 | 1 | 1 | A | het |
| 9457C | 13 | 80 | 6 | 108 | 7 | 55 | 12 | 18 | 15 | C | het |
| 9430A | 8 | 5 | 5 | 9 | 2 | 6 | 5 | 1 | 1 | A | het |
| 9428-2 | 2 | 5 | 5 | 9 | 2 | 6 | 5 | 1 | 1 | A | het |
| 9428 | 2 | 5 | 5 | 9 | 2 | 6 | 5 | 1 | 1 | A | het |
| 9427B | 21 | 17 | 21 | 19 | 27 | 83 | 22 | 7 | 12 | B | het |
| 9414A | 2 | 3 | 90 | 9 | 2 | 6 | 5 | 1 | 1 | A | het |
| 9382 | 2 | 5 | 5 | 9 | 2 | 6 | 5 | 1 | 1 | A | hom$\alpha$ |
| 9358-2 | 2 | 3 | 90 | 9 | 2 | 6 | 5 | 1 | 1 | A | het |
| 9358 | 2 | 3 | 90 | 9 | 2 | 6 | 5 | 1 | 1 | A | het |

| Continuation of Table S1 | | | | | | | | | | | |
|---|---|---|---|---|---|---|---|---|---|---|---|
| Strain | AAT1a | ACC1 | ADP1 | MPIb | SYA1 | VPS13 | ZWF1b | Eburst cluster | MLSTClade | ABC Type | MTL type |
| 9288A | 2 | 3 | 90 | 9 | 2 | 6 | 5 | 1 | 1 | A | het |
| 9263A | 2 | 5 | 5 | 9 | 2 | 6 | 5 | 1 | 1 | A | het |
| 9244B | 1 | 7 | 15 | 6 | 61 | 105 | 112 | 22 | 3 | B | het |
| 9226A | 2 | 5 | 5 | 9 | 2 | 6 | 5 | 1 | 1 | A | het |
| 9186A | 2 | 3 | 90 | 9 | 2 | 6 | 5 | 1 | 1 | A | het |
| 9086A | 2 | 5 | 5 | 9 | 2 | 6 | 5 | 1 | 1 | A | het |
| 9082-2 | 2 | 3 | 90 | 9 | 2 | 6 | 5 | 1 | 1 | A | het |
| 9082 | 2 | 3 | 90 | 9 | 2 | 6 | 5 | 1 | 1 | A | het |
| 9075A | 2 | 5 | 5 | 9 | 2 | 6 | 5 | 1 | 1 | A | het |
| 9071-2 | 2 | 3 | 90 | 9 | 2 | 6 | 5 | 1 | 1 | A | het |
| 9054-3 | 2 | 5 | 5 | 9 | 2 | 6 | 5 | 1 | 1 | A | het |
| 9054-2 | 2 | 5 | 5 | 9 | 2 | 6 | 5 | 1 | 1 | A | het |
| 9054 | 2 | 5 | 5 | 9 | 2 | 6 | 5 | 1 | 1 | A | het |
| 9053C | 9 | 13 | 4 | 14 | 28 | 37 | 18 | 36 | 8 | C | het |
| 9041-3 | 2 | 3 | 90 | 9 | 2 | 6 | 5 | 1 | 1 | A | het |
| 9041 | 2 | 3 | 90 | 9 | 2 | 6 | 5 | 1 | 1 | A | het |
| 9028C | 80 | 10 | 6 | 4 | 30 | 269 | 15 | S | 100* | C | het |
| 8906B | 13 | 10 | 15 | 6 | 7 | 37 | 15 | 4 | 3 | B | het |
| 8896B | 3 | 3 | 21 | 50 | 27 | 109 | 13 | 15 | 14 | B | het |
| 8836-3 | 2 | 5 | 5 | 9 | 2 | 24 | 5 | 1 | 1 | A | het |
| 8836 | 2 | 3 | 90 | 9 | 2 | 6 | 5 | 1 | 1 | A | het |
| 8811-2 | 2 | 3 | 90 | 9 | 2 | 6 | 5 | 1 | 1 | A | het |
| 8811 | 2 | 3 | 90 | 9 | 2 | 6 | 5 | 1 | 1 | A | het |
| 8807-2 | 25 | 7 | 6 | 3 | 6 | 27 | 37 | 5 | 8 | A | het |
| 8807 | 25 | 7 | 6 | 3 | 6 | 27 | 37 | 5 | 8 | A | het |
| 8796-2 | 2 | 3 | 90 | 9 | 2 | 6 | 5 | 1 | 1 | A | het |
| 8796 | 2 | 3 | 90 | 9 | 2 | 6 | 5 | 1 | 1 | A | het |
| 8780-3 | 2 | 3 | 90 | 9 | 2 | 6 | 5 | 1 | 1 | A | het |
| 8780 | 2 | 3 | 90 | 9 | 2 | 6 | 5 | 1 | 1 | A | het |
| 8769-3 | 2 | 2 | 5 | 2 | 2 | 6 | 5 | 1 | 1 | A | het |
| 8769 | 2 | 2 | 5 | 2 | 2 | 6 | 5 | 1 | 1 | A | het |
| 8766A | 2 | 5 | 5 | 9 | 2 | 6 | 5 | 1 | 1 | A | het |
| 8751C | 74 | 14 | 6 | 4 | 30 | 112 | 8 | 125 | 4 | C | het |
| 8737B | 6 | 3 | 21 | 50 | 27 | 109 | 13 | 15 | 14 | B | het |
| 8633-3 | 2 | 5 | 5 | 9 | 2 | 24 | 5 | 1 | 1 | A | het |
| 8633 | 2 | 28 | 21 | 2 | 7 | 289 | 15 | S | 100* | C | het |
| 8574B | 1 | 7 | 15 | 6 | 61 | 105 | 112 | 22 | 3 | B | het |
| 8564B | 59 | 7 | 21 | 2 | 81 | 108 | 90 | 32 | 17 | B | het |
| 8563A | 2 | 5 | 5 | 9 | 2 | 6 | 5 | 1 | 1 | A | het |
| 8558-2 | 21 | 46 | 21 | 28 | 219 | 290 | 5 | S | 17 | B | het |
| 8558 | 21 | 46 | 21 | 28 | 219 | 290 | 5 | S | 17 | B | het |
| 8500A | 2 | 78 | 5 | 9 | 2 | 24 | 5 | 1 | 1 | A | het |
| 8479-2 | 2 | 3 | 90 | 9 | 2 | 6 | 19 | 1 | 1 | A | het |
| 8479 | 2 | 3 | 90 | 9 | 2 | 6 | 19 | 1 | 1 | A | het |
| 8471C | 24 | 7 | 6 | 3 | 6 | 27 | 37 | 5 | 8 | C | het |
| 8437B | 6 | 3 | 21 | 50 | 27 | 109 | 13 | 15 | 14 | B | het |
| 8436-3 | 2 | 3 | 90 | 9 | 2 | 6 | 5 | 1 | 1 | A | het |

| Continuation of Table S1 | | | | | | | | | | | |
|---|---|---|---|---|---|---|---|---|---|---|---|
| Strain | AAT1a | ACC1 | ADP1 | MPIb | SYA1 | VPS13 | ZWF1b | Eburst cluster | MLSTClade | ABC Type | MTL type |
| 8436-2 | 2 | 3 | 90 | 9 | 2 | 6 | 5 | 1 | 1 | A | het |
| 8433-2 | 2 | 3 | 90 | 9 | 2 | 6 | 5 | 1 | 1 | A | het |
| 8433 | 2 | 3 | 90 | 9 | 2 | 6 | 5 | 1 | 1 | A | het |
| 8431-2 | 2 | 3 | 90 | 9 | 2 | 6 | 5 | 1 | 1 | A | het |
| 8431 | 2 | 3 | 90 | 9 | 2 | 6 | 5 | 1 | 1 | A | het |
| 8419-3 | 2 | 3 | 90 | 9 | 2 | 6 | 5 | 1 | 1 | A | het |
| 8419 | 2 | 3 | 90 | 9 | 2 | 6 | 5 | 1 | 1 | A | het |
| 8389-2 | 2 | 3 | 90 | 9 | 2 | 6 | 5 | 1 | 1 | A | het |
| 8389 | 2 | 3 | 90 | 9 | 2 | 6 | 5 | 1 | 1 | A | het |
| 8361-4 | 2 | 3 | 90 | 9 | 2 | 6 | 5 | 1 | 1 | A | het |
| 8361-2 | 2 | 5 | 5 | 9 | 2 | 6 | 5 | 1 | 1 | A | het |
| 8349-2 | 2 | 3 | 90 | 9 | 2 | 6 | 5 | 1 | 1 | A | het |
| 8349 | 2 | 3 | 90 | 9 | 2 | 6 | 5 | 1 | 1 | A | het |
| 8347B | 6 | 3 | 21 | 50 | 27 | 109 | 13 | 15 | 14 | B | het |
| 8311C | 28 | 10 | 21 | 1 | 42 | 11 | 15 | 6 | 11 | C | het |
| 8311-4 | 60 | 10 | 21 | 1 | 42 | 11 | 15 | 6 | 11 | A | het |
| 8311 | 60 | 10 | 21 | 1 | 42 | 11 | 15 | 6 | 11 | A | het |
| 8288-2 | 2 | 3 | 90 | 9 | 2 | 6 | 5 | 1 | 1 | A | het |
| 8288 | 2 | 3 | 90 | 9 | 2 | 6 | 5 | 1 | 1 | A | het |
| 8285A | 2 | 5 | 5 | 9 | 2 | 24 | 5 | 1 | 1 | A | het |
| 8277A | 2 | 3 | 90 | 9 | 2 | 6 | 5 | 1 | 1 | A | het |
| 8259-2 | 2 | 3 | 90 | 9 | 2 | 6 | 5 | 1 | 1 | A | het |
| 8259 | 2 | 5 | 5 | 9 | 2 | 6 | 5 | 1 | 1 | A | het |
| 8258-2 | 2 | 5 | 5 | 9 | 2 | 24 | 5 | 1 | 1 | A | het |
| 8258 | 2 | 5 | 5 | 9 | 2 | 24 | 5 | 1 | 1 | A | het |
| 8256-2 | 2 | 3 | 90 | 9 | 2 | 6 | 5 | 1 | 1 | A | het |
| 8256 | 2 | 3 | 90 | 9 | 2 | 6 | 5 | 1 | 1 | A | het |
| 8232C | 28 | 7 | 6 | 1 | 2 | 11 | 15 | 136 | 11 | C | het |
| 8230-2 | 2 | 5 | 5 | 9 | 2 | 6 | 5 | 1 | 1 | A | het |
| 8230 | 2 | 5 | 5 | 9 | 2 | 6 | 5 | 1 | 1 | A | het |
| 8221B | 6 | 3 | 21 | 50 | 27 | 109 | 13 | 15 | 14 | B | het |
| 8198C | 1 | 3 | 4 | 3 | 7 | 45 | 112 | 21 | 8 | C | het |
| 8182B | 60 | 13 | 6 | 1 | 7 | 11 | 15 | 6 | 11 | B | het |
| 8166-2 | 2 | 78 | 5 | 9 | 2 | 6 | 5 | 1 | 1 | A | het |
| 8166 | 2 | 78 | 5 | 9 | 2 | 6 | 5 | 1 | 1 | A | het |
| 8149B | 80 | 3 | 6 | 4 | 31 | 45 | 13 | 25 | 14 | B | het |
| 8134B | 6 | 3 | 21 | 50 | 27 | 109 | 13 | 15 | 14 | B | het |
| 8133-3 | 80 | 7 | 6 | 2 | 2 | 288 | 112 | S | 101* | A | het |
| 8133 | 2 | 5 | 5 | 9 | 2 | 6 | 5 | 1 | 1 | A | het |
| 8115B | 59 | 5 | 21 | 2 | 80 | 108 | 15 | 13 | 17 | B | het |
| 5290A | 2 | 3 | 90 | 9 | 2 | 6 | 5 | 1 | 1 | A | het |
| 5266A | 2 | 5 | 5 | 9 | 2 | 24 | 5 | 1 | 1 | A | het |
| 5257B | 13 | 80 | 6 | 108 | 7 | 105 | 12 | 18 | 15 | B | het |
| 5255A | 2 | 5 | 5 | 9 | 2 | 6 | 5 | 1 | 1 | A | het |
| 5250B | 13 | 80 | 6 | 108 | 7 | 105 | 12 | 18 | 15 | B | het |
| 5241A | 2 | 3 | 90 | 9 | 2 | 6 | 5 | 1 | 1 | A | het |
| 5237A | 2 | 3 | 90 | 9 | 2 | 6 | 5 | 1 | 1 | A | het |

Continuation of Table S1

| Strain | AAT1a | ACC1 | ADP1 | MPIb | SYA1 | VPS13 | ZWF1b | Eburst cluster | MLSTClade | ABC Type | MTL type |
|---|---|---|---|---|---|---|---|---|---|---|---|
| 5210A | 2 | 3 | 90 | 9 | 2 | 6 | 5 | 1 | 1 | A | het |
| 5200A | 2 | 5 | 5 | 9 | 2 | 6 | 5 | 1 | 1 | A | het |
| 5199A | 2 | 5 | 5 | 9 | 2 | 6 | 5 | 1 | 1 | A | het |
| 5146A | 2 | 5 | 5 | 9 | 2 | 6 | 5 | 1 | 1 | A | het |
| 5142A | 2 | 3 | 90 | 9 | 2 | 6 | 5 | 1 | 1 | A | het |
| 5132C | 25 | 7 | 6 | 3 | 6 | 27 | 37 | 5 | 8 | C | het |
| 5080A | 2 | 2 | 5 | 9 | 2 | 6 | 5 | 1 | 1 | A | het |
| 5079C | 25 | 7 | 6 | 9 | 6 | 27 | 37 | 5 | 8 | C | het |
| 5053A | 2 | 3 | 90 | 9 | 2 | 6 | 5 | 1 | 1 | A | het |
| 5049B | 59 | 5 | 21 | 2 | 80 | 108 | 15 | 13 | 17 | B | het |
| 5045A | 2 | 78 | 5 | 9 | 2 | 24 | 5 | 1 | 1 | A | het |
| 5028C | 55 | 14 | 4 | 3 | 6 | 45 | 15 | 5 | 8 | C | het |
| 2284C | 25 | 7 | 6 | 3 | 6 | 27 | 37 | 5 | 8 | C | het |
| 2252C | 1 | 3 | 4 | 3 | 7 | 73 | 112 | 21 | 8 | C | het |
| 2206B | 6 | 3 | 6 | 4 | 53 | 109 | 13 | 15 | 14 | B | het |
| 1131B | 59 | 5 | 21 | 2 | 80 | 108 | 15 | 13 | 17 | B | het |
| 1112C | 60 | 7 | 21 | 1 | 7 | 11 | 15 | 6 | 11 | C | het |
| 1035C | 1 | 3 | 4 | 3 | 211 | 73 | 112 | 21 | 8 | C | het |
| 1034B | 6 | 3 | 21 | 50 | 27 | 109 | 13 | 15 | 14 | B | het |
| 1021B | 6 | 3 | 6 | 4 | 53 | 109 | 13 | 15 | 14 | B | het |
| 9071 | 2 | 3 | 90 | 9 | 2 | 6 | 5 | 1 | 1 | A | het |
| 8508 | 2 | 3 | 90 | 9 | 2 | 6 | 5 | 1 | 1 | A | hom$\alpha$ |

End of Table S1

**Table S2.** The MLST of C. albicans strains isolated from the subsequent samples of treatment failure VVC patients. The digit after P is the identifier of the patient, and the digit after – is the number of times she was resampled.

| Strain | AAT1a | ACC1 | ADP1 | MPIb | SYA1 | VPS13 | ZWF1b | Eburst | MLST Clade |
|---|---|---|---|---|---|---|---|---|---|
| P005 | 55 | 14 | 4 | 3 | 6 | 45 | 15 | 5 | 8 |
| P005-3 | 55 | 14 | 4 | 3 | 6 | 45 | 15 | 5 | 8 |
| P005-4 | 55 | 14 | 4 | 3 | 6 | 45 | 15 | 5 | 8 |
| P014 | 2 | 5 | 5 | 9 | 2 | 6 | 5 | 1 | 1 |
| P014-2 | 2 | 5 | 5 | 9 | 2 | 6 | 5 | 1 | 1 |
| P014-3 | 2 | 5 | 5 | 9 | 2 | 6 | 5 | 1 | 1 |
| P015 | 2 | 5 | 5 | 9 | 2 | 6 | 5 | 1 | 1 |
| P015-2 | 2 | 5 | 5 | 9 | 2 | 6 | 5 | 1 | 1 |
| P019 | 13 | 26 | 5 | 3 | 93 | 53 | 12 | 29 | 1 |
| P019-2 | 13 | 26 | 5 | 3 | 93 | 53 | 12 | 29 | 1 |
| P019-3 | 13 | 26 | 5 | 3 | 93 | 53 | 12 | 29 | 1 |
| P028 | 2 | 5 | 5 | 9 | 2 | 6 | 5 | 1 | 1 |
| P028-2 | 2 | 5 | 5 | 9 | 2 | 6 | 5 | 1 | 1 |
| P040 | 2 | 3 | 90 | 9 | 2 | 6 | 5 | 1 | 1 |
| P040-2 | 2 | 5 | 90 | 9 | 2 | 6 | 5 | 1 | 1 |
| P044 | 2 | 5 | 5 | 9 | 2 | 6 | 5 | 1 | 1 |
| P044-2 | 2 | 3 | 5 | 9 | 2 | 6 | 5 | 1 | 1 |
| P044-3 | 2 | 5 | 5 | 9 | 2 | 6 | 5 | 1 | 1 |
| P044-4 | 2 | 5 | 5 | 9 | 2 | 6 | 5 | 1 | 1 |
| P044-5 | 2 | 5 | 5 | 9 | 2 | 6 | 5 | 1 | 1 |
| P044-6 | 2 | 5 | 5 | 9 | 2 | 6 | 5 | 1 | 1 |
| P044-7 | 2 | 5 | 5 | 9 | 2 | 6 | 5 | 1 | 1 |
| P044-9 | 2 | 5 | 5 | 9 | 2 | 6 | 5 | 1 | 1 |
| P047 | 2 | 5 | 5 | 9 | 2 | 24 | 5 | 1 | 1 |
| P047-2 | 2 | 5 | 5 | 9 | 2 | 24 | 5 | 1 | 1 |
| P047-3 | 2 | 5 | 5 | 9 | 2 | 24 | 5 | 1 | 1 |
| P049 | 2 | 5 | 5 | 9 | 2 | 6 | 5 | 1 | 1 |
| P049-2 | 2 | 5 | 5 | 9 | 2 | 6 | 5 | 1 | 1 |
| P050 | 2 | 3 | 90 | 9 | 2 | 6 | 5 | 1 | 1 |
| P050-2 | 2 | 3 | 90 | 9 | 2 | 6 | 5 | 1 | 1 |
| P050-3 | 2 | 3 | 90 | 9 | 2 | 6 | 5 | 1 | 1 |
| P054 | 2 | 5 | 5 | 9 | 2 | 6 | 5 | 1 | 1 |
| P054-2 | 2 | 5 | 5 | 9 | 2 | 6 | 5 | 1 | 1 |
| P060 | 5 | 10 | 37 | 4 | 34 | 105 | 22 | 133 | 3 |
| P060-2 | 5 | 10 | 37 | 4 | 34 | 105 | 22 | 133 | 3 |
| P065 | 2 | 5 | 5 | 9 | 2 | 6 | 5 | 1 | 1 |
| P065-2 | 2 | 5 | 5 | 9 | 2 | 6 | 5 | 1 | 1 |
| P065-3 | 2 | 5 | 5 | 9 | 2 | 6 | 5 | 1 | 1 |
| P067 | 8 | 5 | 5 | 9 | 2 | 6 | 5 | 1 | 1 |
| P067-2 | 8 | 5 | 5 | 9 | 2 | 6 | 5 | 1 | 1 |

Table S3: The MICs of 14 different drugs were determined for each C. albicans isolate.

| Begin of Table S3 | | | | | | | | | | | | | | | |
|---|---|---|---|---|---|---|---|---|---|---|---|---|---|---|---|
| Strain No. | Clade | BUC | CLO | MIC | FLC | TEC | VRC | ITC | AMB | NYS | AFG | CFG | MFG | 5FC | TRB |
| 5045 | 1 | 0.030 | 0.030 | 0.125 | 0.125 | 0.030 | 0.030 | 0.030 | 0.125 | 4.000 | 0.015 | 0.250 | 0.030 | 0.500 | 256.000 |
| 5053 | 1 | 0.250 | 0.060 | 0.250 | 2.000 | 0.250 | 0.125 | 0.250 | 0.125 | 0.500 | 0.015 | 0.500 | 16.000 | 4.000 | 256.000 |
| 5080 | 1 | 0.250 | 0.030 | 0.250 | 1.000 | 0.250 | 0.030 | 0.250 | 0.250 | 0.125 | 0.015 | 0.250 | 0.015 | 8.000 | 256.000 |
| 5142 | 1 | 1.000 | 0.060 | 0.250 | 1.000 | 0.250 | 0.250 | 0.030 | 0.500 | 0.500 | 0.015 | 0.250 | 0.008 | 1.000 | 256.000 |
| 5146 | 1 | 0.060 | 0.030 | 0.060 | 0.250 | 0.030 | 0.030 | 0.030 | 0.125 | 4.000 | 0.015 | 0.500 | 0.060 | 1.000 | 256.000 |

| Continuation of Table S3 | | | | | | | | | | | | | | | |
|---|---|---|---|---|---|---|---|---|---|---|---|---|---|---|---|
| Strain No. | Clade | BUC | CLO | MIC | FLC | TEC | VRC | ITC | AMB | NYS | AFG | CFG | MFG | 5FC | TRB |
| 5199 | 1 | 0.125 | 0.030 | 0.030 | 0.125 | 0.030 | 0.030 | 0.030 | 0.250 | 4.000 | 0.015 | 0.250 | 0.125 | 1.000 | 256.000 |
| 5200 | 1 | 0.030 | 0.015 | 0.060 | 0.250 | 0.030 | 0.030 | 0.060 | 0.250 | 0.500 | 0.015 | 0.500 | 1.000 | 1.000 | 256.000 |
| 5210 | 1 | 0.060 | 0.030 | 0.250 | 2.000 | 0.500 | 0.125 | 0.030 | 0.250 | 4.000 | 0.015 | 0.250 | 16.000 | 4.000 | 256.000 |
| 5237 | 1 | 1.000 | 0.125 | 0.250 | 4.000 | 1.000 | 0.250 | 0.030 | 0.250 | 0.500 | 0.015 | 0.500 | 0.008 | 1.000 | 256.000 |
| 5241 | 1 | 0.500 | 0.030 | 0.250 | 1.000 | 0.250 | 0.125 | 0.030 | 0.250 | 4.000 | 0.015 | 0.250 | 2.000 | 1.000 | 256.000 |
| 5255 | 1 | 0.250 | 0.030 | 0.030 | 0.125 | 0.030 | 0.030 | 0.030 | 0.500 | 0.500 | 0.015 | 0.250 | 0.060 | 1.000 | 256.000 |
| 5266 | 1 | 0.500 | 0.030 | 0.030 | 0.500 | 0.030 | 0.030 | 0.030 | 0.250 | 2.000 | 0.015 | 0.250 | 0.500 | 1.000 | 128.000 |
| 5290 | 1 | 0.500 | 0.030 | 0.030 | 0.500 | 0.030 | 0.030 | 0.030 | 0.125 | 2.000 | 0.015 | 0.250 | 0.250 | 1.000 | 128.000 |
| 8133 | 1 | 0.030 | 0.030 | 0.030 | 0.500 | 0.030 | 0.030 | 0.030 | 0.060 | 4.000 | 0.015 | 0.250 | 0.060 | 2.000 | 256.000 |
| 8166 | 1 | 0.030 | 0.030 | 0.125 | 2.000 | 0.500 | 0.030 | 0.030 | 0.250 | 0.500 | 0.015 | 0.250 | 0.008 | 64.000 | 256.000 |
| 2288643 | 1 | 0.125 | 0.030 | 0.125 | 1.000 | 0.500 | 0.125 | 0.030 | 0.250 | 0.500 | 0.015 | 0.250 | 0.500 | 64.000 | 128.000 |
| 8230 | 1 | 0.500 | 0.030 | 0.060 | 1.000 | 0.500 | 0.500 | 0.030 | 0.250 | 0.500 | 0.015 | 0.500 | 0.008 | 1.000 | 128.000 |
| 2312018 | 1 | 0.500 | 0.030 | 0.060 | 1.000 | 0.250 | 0.250 | 0.030 | 0.060 | 0.500 | 0.015 | 0.500 | 0.008 | 1.000 | 256.000 |
| 8256 | 1 | 0.500 | 0.125 | 0.250 | 4.000 | 2.000 | 0.250 | 0.250 | 0.500 | 0.500 | 0.015 | 0.250 | 2.000 | 2.000 | 256.000 |
| 2321514 | 1 | 0.500 | 0.125 | 0.250 | 2.000 | 0.250 | 0.125 | 0.030 | 0.500 | 0.500 | 0.015 | 0.250 | 2.000 | 2.000 | 256.000 |
| 8258 | 1 | 0.125 | 0.030 | 0.060 | 0.500 | 0.030 | 0.030 | 0.030 | 0.250 | 0.500 | 0.015 | 0.250 | 0.250 | 1.000 | 256.000 |
| 2322245 | 1 | 0.125 | 0.030 | 0.060 | 0.500 | 0.030 | 0.030 | 0.030 | 0.060 | 0.500 | 0.015 | 0.500 | 0.500 | 0.250 | 256.000 |
| 8259 | 1 | 0.030 | 0.030 | 0.015 | 2.000 | 0.030 | 0.030 | 0.030 | 0.060 | 0.500 | 0.015 | 0.250 | 0.015 | 0.125 | 256.000 |
| 2322610 | 1 | 0.030 | 0.030 | 0.060 | 2.000 | 0.125 | 0.125 | 0.030 | 0.060 | 0.500 | 0.015 | 0.500 | 0.500 | 1.000 | 256.000 |
| 8277 | 1 | 0.060 | 0.125 | 0.030 | 1.000 | 0.500 | 0.500 | 0.125 | 0.500 | 0.500 | 0.015 | 0.250 | 0.015 | 1.000 | 256.000 |
| 8285 | 1 | 0.030 | 0.030 | 0.030 | 1.000 | 0.030 | 0.030 | 0.125 | 0.125 | 4.000 | 0.015 | 0.250 | 0.015 | 0.125 | 256.000 |
| 8288 | 1 | 0.500 | 0.500 | 0.500 | 8.000 | 0.500 | 0.500 | 1.000 | 0.125 | 0.500 | 0.015 | 0.500 | 0.008 | 1.000 | 256.000 |
| 2333202 | 1 | 0.250 | 1.000 | 1.000 | 8.000 | 1.000 | 0.500 | 0.030 | 0.250 | 0.500 | 0.015 | 0.500 | 0.008 | 1.000 | 256.000 |
| 8349 | 1 | 0.060 | 0.060 | 0.125 | 2.000 | 0.250 | 0.125 | 0.030 | 0.060 | 0.500 | 0.015 | 0.250 | 0.008 | 1.000 | 256.000 |
| 2355482 | 1 | 0.060 | 0.030 | 0.250 | 2.000 | 1.000 | 0.060 | 0.030 | 0.500 | 0.500 | 0.015 | 0.500 | 1.000 | 1.000 | 256.000 |
| 2359865 | 1 | 2.000 | 0.030 | 0.250 | 2.000 | 2.000 | 1.000 | 0.030 | 0.125 | 0.500 | 0.015 | 0.500 | 1.000 | 2.000 | 256.000 |
| 2359924 | 1 | 0.125 | 0.060 | 0.250 | 2.000 | 0.500 | 0.125 | 0.030 | 0.250 | 0.500 | 0.015 | 0.250 | 0.008 | 2.000 | 256.000 |
| 8389 | 1 | 0.125 | 0.125 | 0.015 | 2.000 | 0.500 | 0.125 | 0.030 | 0.250 | 0.500 | 0.015 | 0.250 | 1.000 | 2.000 | 256.000 |
| 2370092 | 1 | 0.500 | 0.125 | 0.500 | 2.000 | 0.500 | 0.125 | 0.030 | 0.250 | 0.500 | 0.015 | 0.250 | 0.008 | 2.000 | 256.000 |
| 8419 | 1 | 0.125 | 0.060 | 0.250 | 2.000 | 0.250 | 0.125 | 0.030 | 0.250 | 0.500 | 0.015 | 0.250 | 0.008 | 2.000 | 256.000 |
| 2381077 | 1 | 0.125 | 0.060 | 0.015 | 2.000 | 0.250 | 0.060 | 0.030 | 0.250 | 0.500 | 0.015 | 0.250 | 0.008 | 2.000 | 256.000 |
| 8431 | 1 | 0.060 | 0.030 | 0.125 | 2.000 | 0.250 | 0.125 | 0.030 | 0.060 | 0.500 | 0.015 | 0.250 | 0.008 | 1.000 | 256.000 |
| 2385432 | 1 | 0.125 | 0.030 | 0.250 | 2.000 | 1.000 | 0.125 | 0.030 | 0.250 | 4.000 | 0.015 | 0.500 | 1.000 | 1.000 | 256.000 |
| 8433 | 1 | 0.250 | 0.125 | 0.015 | 2.000 | 0.250 | 0.060 | 0.030 | 0.250 | 0.500 | 0.015 | 0.250 | 0.500 | 1.000 | 256.000 |
| 2386163 | 1 | 0.125 | 0.125 | 0.015 | 2.000 | 0.250 | 0.060 | 0.030 | 0.250 | 0.500 | 0.015 | 0.250 | 0.008 | 1.000 | 256.000 |
| 2387258 | 1 | 0.125 | 0.250 | 0.015 | 1.000 | 0.500 | 0.250 | 0.500 | 0.500 | 0.500 | 0.008 | 0.250 | 0.008 | 1.000 | 256.000 |
| 2387287 | 1 | 0.500 | 0.060 | 0.015 | 2.000 | 0.250 | 0.250 | 0.060 | 0.500 | 0.500 | 0.015 | 0.250 | 0.008 | 1.000 | 256.000 |
| 8479 | 1 | 0.125 | 0.060 | 0.125 | 1.000 | 0.500 | 0.060 | 0.030 | 0.250 | 0.500 | 0.015 | 0.250 | 0.008 | 1.000 | 256.000 |
| 2402964 | 1 | 0.125 | 0.250 | 0.125 | 2.000 | 0.125 | 0.060 | 0.030 | 0.250 | 0.500 | 0.015 | 0.250 | 1.000 | 2.000 | 256.000 |
| 8500 | 1 | 0.060 | 0.030 | 0.030 | 1.000 | 0.030 | 0.030 | 0.030 | 0.500 | 0.500 | 0.015 | 0.125 | 0.015 | 1.000 | 256.000 |
| 8563 | 1 | 0.250 | 0.030 | 0.030 | 0.250 | 0.030 | 0.030 | 0.060 | 0.250 | 0.500 | 0.015 | 0.125 | 0.030 | 1.000 | 256.000 |
| 2459239 | 1 | 0.125 | 0.030 | 0.030 | 0.500 | 0.030 | 0.030 | 0.030 | 0.125 | 4.000 | 0.015 | 0.500 | 0.125 | 2.000 | 128.000 |
| 8766 | 1 | 0.030 | 0.060 | 0.030 | 1.000 | 0.060 | 0.030 | 0.030 | 1.000 | 0.500 | 0.015 | 0.250 | 1.000 | 1.000 | 256.000 |
| 8769 | 1 | 0.030 | 0.030 | 0.015 | 0.250 | 0.030 | 0.030 | 0.030 | 0.500 | 0.500 | 0.015 | 0.500 | 0.060 | 0.125 | 128.000 |
| 2508912 | 1 | 0.060 | 0.030 | 0.030 | 0.250 | 0.030 | 0.030 | 0.030 | 0.500 | 0.500 | 0.015 | 0.250 | 0.015 | 0.500 | 32.000 |
| 8780 | 1 | 0.250 | 0.060 | 0.125 | 2.000 | 0.250 | 0.060 | 0.030 | 0.250 | 0.500 | 0.015 | 0.250 | 0.008 | 1.000 | 256.000 |
| 2512930 | 1 | 0.030 | 0.030 | 0.060 | 1.000 | 0.250 | 0.060 | 0.030 | 0.060 | 4.000 | 0.015 | 0.500 | 1.000 | 2.000 | 256.000 |

| Continuation of Table S3 | | | | | | | | | | | | | | | |
|---|---|---|---|---|---|---|---|---|---|---|---|---|---|---|---|
| Strain No. | Clade | BUC | CLO | MIC | FLC | TEC | VRC | ITC | AMB | NYS | AFG | CFG | MFG | 5FC | TRB |
| 8796 | 1 | 0.125 | 0.060 | 0.060 | 2.000 | 0.250 | 0.125 | 0.030 | 0.060 | 0.500 | 0.015 | 0.500 | 0.008 | 2.000 | 256.000 |
| 2518745 | 1 | 0.060 | 0.060 | 0.250 | 2.000 | 0.250 | 0.250 | 0.030 | 0.060 | 0.500 | 0.015 | 0.250 | 0.008 | 2.000 | 256.000 |
| 8811 | 1 | 0.030 | 0.125 | 0.125 | 1.000 | 0.250 | 0.125 | 0.030 | 0.060 | 0.500 | 0.015 | 0.250 | 0.008 | 2.000 | 256.000 |
| 2524224 | 1 | 0.125 | 0.125 | 0.125 | 2.000 | 0.060 | 0.060 | 0.030 | 0.500 | 8.000 | 0.015 | 0.500 | 0.500 | 4.000 | 256.000 |
| 8836 | 1 | 0.125 | 0.030 | 0.125 | 2.000 | 0.250 | 0.060 | 0.030 | 0.250 | 0.500 | 0.015 | 0.250 | 0.008 | 2.000 | 256.000 |
| 2533384 | 1 | 0.500 | 0.030 | 0.030 | 1.000 | 0.030 | 0.030 | 0.030 | 0.500 | 8.000 | 0.015 | 0.500 | 0.250 | 2.000 | 128.000 |
| 9041 | 1 | 0.500 | 0.125 | 0.500 | 1.000 | 0.250 | 0.060 | 0.125 | 0.500 | 0.500 | 0.015 | 0.250 | 0.008 | 1.000 | 256.000 |
| 2608258 | 1 | 0.060 | 0.030 | 0.125 | 2.000 | 0.500 | 0.125 | 0.030 | 0.060 | 0.500 | 0.015 | 0.500 | 0.008 | 2.000 | 256.000 |
| 9054 | 1 | 0.500 | 0.030 | 0.015 | 2.000 | 4.000 | 4.000 | 0.030 | 0.125 | 0.500 | 0.015 | 0.500 | 4.000 | 2.000 | 128.000 |
| 2612978 | 1 | 8.000 | 0.250 | 0.015 | 1.000 | 0.250 | 0.060 | 0.030 | 0.500 | 0.500 | 0.015 | 0.500 | 0.008 | 1.000 | 256.000 |
| 2613006 | 1 | 0.500 | 0.030 | 0.015 | 1.000 | 2.000 | 0.250 | 0.500 | 0.500 | 0.500 | 0.015 | 0.250 | 0.008 | 1.000 | 256.000 |
| 9071 | 1 | 0.250 | 0.030 | 0.125 | 2.000 | 0.250 | 0.060 | 0.030 | 0.060 | 4.000 | 0.015 | 0.250 | 1.000 | 1.000 | 256.000 |
| 2619187 | 1 | 0.250 | 0.125 | 0.015 | 1.000 | 0.250 | 0.125 | 0.060 | 0.125 | 0.500 | 0.015 | 0.500 | 1.000 | 1.000 | 256.000 |
| 9075 | 1 | 2.000 | 0.030 | 0.030 | 1.000 | 1.000 | 2.000 | 0.030 | 0.125 | 2.000 | 0.015 | 0.500 | 8.000 | 4.000 | 256.000 |
| 9082 | 1 | 0.250 | 0.030 | 0.250 | 2.000 | 0.250 | 0.125 | 0.030 | 0.060 | 0.500 | 0.015 | 0.500 | 2.000 | 1.000 | 256.000 |
| 2623205 | 1 | 0.060 | 0.030 | 0.060 | 2.000 | 0.250 | 0.060 | 0.030 | 0.060 | 0.500 | 0.015 | 0.500 | 0.500 | 1.000 | 256.000 |
| 9086 | 1 | 0.030 | 0.030 | 0.030 | 1.000 | 0.030 | 0.030 | 0.030 | 1.000 | 0.500 | 0.015 | 0.250 | 0.030 | 0.125 | 256.000 |
| 9186 | 1 | 2.000 | 0.250 | 0.030 | 2.000 | 2.000 | 0.125 | 0.250 | 0.500 | 0.500 | 0.015 | 0.250 | 1.000 | 1.000 | 256.000 |
| 9226 | 1 | 0.125 | 0.030 | 0.030 | 1.000 | 0.500 | 0.250 | 0.030 | 0.060 | 0.500 | 0.015 | 0.250 | 1.000 | 1.000 | 256.000 |
| 9263 | 1 | 0.060 | 0.030 | 0.030 | 0.125 | 0.030 | 0.030 | 0.030 | 0.060 | 2.000 | 0.015 | 0.250 | 0.060 | 0.125 | 128.000 |
| 9288 | 1 | 1.000 | 0.125 | 0.030 | 1.000 | 0.500 | 0.500 | 0.500 | 0.250 | 0.500 | 0.015 | 0.125 | 1.000 | 1.000 | 256.000 |
| 9358 | 1 | 0.125 | 0.030 | 0.125 | 1.000 | 0.250 | 0.060 | 0.030 | 0.125 | 0.500 | 0.015 | 0.005 | 1.000 | 1.000 | 256.000 |
| 2724012 | 1 | 0.030 | 0.060 | 0.125 | 2.000 | 0.125 | 0.060 | 0.030 | 0.060 | 0.500 | 0.015 | 0.500 | 0.500 | 1.000 | 256.000 |
| 9414 | 1 | 0.060 | 0.250 | 0.030 | 1.000 | 0.500 | 0.250 | 0.125 | 0.500 | 0.500 | 0.015 | 0.250 | 0.015 | 1.000 | 256.000 |
| 9428 | 1 | 0.125 | 0.030 | 0.060 | 1.000 | 1.000 | 0.250 | 0.030 | 0.125 | 0.500 | 0.015 | 0.500 | 0.008 | 2.000 | 128.000 |
| 9430 | 1 | 4.000 | 0.030 | 0.030 | 1.000 | 2.000 | 1.000 | 0.030 | 0.500 | 4.000 | 0.015 | 0.500 | 4.000 | 1.000 | 256.000 |
| 9464 | 1 | 0.500 | 0.030 | 0.030 | 1.000 | 0.500 | 0.125 | 0.030 | 0.250 | 4.000 | 0.015 | 0.500 | 0.015 | 1.000 | 256.000 |
| 9470 | 1 | 0.250 | 0.030 | 0.030 | 1.000 | 0.125 | 0.060 | 0.030 | 0.060 | 2.000 | 0.015 | 0.250 | 0.015 | 1.000 | 256.000 |
| 9486 | 1 | 2.000 | 0.060 | 0.030 | 2.000 | 1.000 | 1.000 | 0.500 | 0.500 | 0.500 | 0.015 | 0.125 | 1.000 | 1.000 | 256.000 |
| 9497 | 1 | 0.125 | 0.500 | 0.125 | 2.000 | 0.250 | 0.125 | 0.125 | 0.125 | 0.500 | 0.015 | 0.500 | 0.008 | 2.000 | 256.000 |
| 2774781 | 1 | 0.250 | 0.060 | 0.015 | 2.000 | 0.250 | 0.060 | 0.030 | 0.125 | 0.500 | 0.015 | 0.500 | 1.000 | 2.000 | 256.000 |
| ATCC90028 | 1 | 0.030 | 0.030 | 0.060 | 0.250 | 0.030 | 0.030 | 0.060 | 0.500 | 0.500 | 0.008 | 0.250 | 0.008 | 0.500 | 251.000 |
| E165 | 1 | 0.250 | 0.030 | 0.060 | 1.000 | 0.250 | 0.250 | 0.030 | 0.125 | 0.500 | 0.015 | 0.500 | 0.008 | 2.000 | 128.000 |
| E188 | 1 | 0.500 | 0.030 | 0.125 | 0.500 | 0.030 | 0.060 | 0.030 | 0.500 | 8.000 | 0.015 | 0.500 | 0.250 | 1.000 | 128.000 |
| E188-10 | 1 | 0.250 | 0.125 | 0.060 | 0.500 | 0.030 | 0.060 | 0.030 | 0.500 | 0.500 | 0.015 | 0.500 | 0.008 | 1.000 | 251.000 |
| E191 | 1 | 0.500 | 0.030 | 0.500 | 4.000 | 1.000 | 0.500 | 0.125 | 0.500 | 0.500 | 0.015 | 0.005 | 0.008 | 2.000 | 256.000 |
| E191-8 | 1 | 1.000 | 0.030 | 0.250 | 4.000 | 1.000 | 0.500 | 0.030 | 0.250 | 1.000 | 0.015 | 0.500 | 0.008 | 2.000 | 256.000 |
| E196 | 1 | 0.500 | 0.030 | 0.250 | 4.000 | 1.000 | 1.000 | 0.030 | 0.125 | 0.500 | 0.015 | 0.500 | 0.008 | 2.000 | 256.000 |
| E196-10 | 1 | 0.500 | 0.030 | 0.500 | 4.000 | 1.000 | 1.000 | 0.030 | 0.500 | 0.500 | 0.015 | 0.500 | 0.008 | 2.000 | 256.000 |
| E235 | 1 | 0.030 | 0.030 | 0.030 | 0.125 | 0.030 | 0.030 | 0.030 | 0.125 | 2.000 | 0.015 | 0.250 | 0.030 | 2.000 | 128.000 |
| E243 | 1 | 0.060 | 0.030 | 0.030 | 0.125 | 0.030 | 0.030 | 0.030 | 0.125 | 2.000 | 0.015 | 0.250 | 0.125 | 0.125 | 64.000 |
| E275 | 1 | 1.000 | 0.030 | 0.125 | 1.000 | 0.125 | 1.000 | 0.030 | 0.250 | 0.500 | 0.015 | 0.500 | 0.008 | 2.000 | 256.000 |
| E275-4 | 1 | 1.000 | 0.030 | 0.060 | 2.000 | 0.250 | 0.250 | 0.030 | 0.125 | 4.000 | 0.015 | 0.500 | 1.000 | 1.000 | 32.000 |
| E277-5 | 1 | 0.500 | 0.030 | 0.500 | 4.000 | 1.000 | 0.500 | 0.030 | 0.125 | 1.000 | 0.015 | 0.500 | 0.008 | 1.000 | 256.000 |
| E286aa | 1 | 2.000 | 0.500 | 16.000 | 8.000 | 1.000 | 0.250 | 1.000 | 0.125 | 0.500 | 0.008 | 0.500 | 0.008 | 4.000 | 251.000 |
| E306 | 1 | 0.250 | 0.250 | 0.250 | 2.000 | 0.250 | 0.060 | 0.030 | 0.500 | 8.000 | 0.015 | 0.250 | 2.000 | 2.000 | 256.000 |
| E306-3 | 1 | 0.030 | 0.060 | 0.125 | 2.000 | 0.125 | 0.125 | 0.030 | 0.125 | 0.500 | 0.015 | 0.500 | 0.008 | 2.000 | 256.000 |

| Continuation of Table S3 | | | | | | | | | | | | | | | |
|---|---|---|---|---|---|---|---|---|---|---|---|---|---|---|---|
| Strain No. | Clade | BUC | CLO | MIC | FLC | TEC | VRC | ITC | AMB | NYS | AFG | CFG | MFG | 5FC | TRB |
| E309 | 1 | 0.250 | 0.500 | 8.000 | 1.000 | 0.250 | 0.125 | 0.250 | 0.125 | 0.500 | 0.008 | 0.500 | 0.008 | 1.000 | 251.000 |
| E309-3 | 1 | 1.000 | 0.030 | 0.250 | 4.000 | 0.500 | 1.000 | 0.500 | 0.125 | 0.500 | 0.015 | 0.500 | 0.008 | 2.000 | 256.000 |
| E315 | 1 | 0.030 | 0.030 | 0.060 | 0.250 | 0.060 | 0.030 | 0.030 | 0.060 | 0.500 | 0.015 | 0.250 | 0.015 | 0.125 | 256.000 |
| E336 | 1 | 0.250 | 0.030 | 0.250 | 4.000 | 1.000 | 0.500 | 0.030 | 0.125 | 0.500 | 0.015 | 0.500 | 2.000 | 2.000 | 256.000 |
| E336-5 | 1 | 2.000 | 0.030 | 0.500 | 4.000 | 1.000 | 1.000 | 0.250 | 0.500 | 0.500 | 0.015 | 0.500 | 0.008 | 2.000 | 256.000 |
| E393 | 1 | 0.500 | 0.060 | 0.250 | 2.000 | 1.000 | 0.125 | 0.030 | 0.500 | 8.000 | 0.015 | 0.500 | 0.500 | 2.000 | 256.000 |
| E393-4 | 1 | 0.250 | 0.030 | 0.250 | 4.000 | 4.000 | 1.000 | 0.030 | 0.125 | 4.000 | 0.015 | 0.500 | 2.000 | 2.000 | 256.000 |
| E393-5 | 1 | 0.250 | 0.125 | 0.250 | 1.000 | 0.125 | 0.125 | 0.125 | 0.500 | 0.500 | 0.015 | 0.250 | 0.008 | 1.000 | 251.000 |
| E423 | 1 | 1.000 | 0.125 | 0.500 | 2.000 | 0.250 | 0.250 | 0.030 | 0.250 | 8.000 | 0.015 | 0.250 | 0.008 | 1.000 | 256.000 |
| E423-4 | 1 | 4.000 | 0.060 | 8.000 | 1.000 | 0.125 | 0.125 | 0.125 | 0.250 | 1.000 | 0.008 | 0.250 | 0.008 | 1.000 | 251.000 |
| E423-5 | 1 | 0.060 | 0.125 | 0.125 | 2.000 | 0.125 | 0.125 | 0.030 | 0.500 | 8.000 | 0.015 | 0.500 | 1.000 | 2.000 | 128.000 |
| E428-6 | 1 | 0.500 | 0.030 | 0.125 | 2.000 | 0.500 | 0.250 | 0.060 | 0.500 | 1.000 | 0.015 | 0.500 | 2.000 | 2.000 | 128.000 |
| E428-7 | 1 | 4.000 | 0.030 | 4.000 | 2.000 | 0.250 | 0.500 | 0.125 | 0.500 | 1.000 | 0.008 | 0.500 | 0.008 | 1.000 | 128.000 |
| E449 | 1 | 0.250 | 0.030 | 0.250 | 8.000 | 4.000 | 0.500 | 0.030 | 0.125 | 4.000 | 0.015 | 0.500 | 2.000 | 2.000 | 128.000 |
| E449-5 | 1 | 0.500 | 0.030 | 0.250 | 4.000 | 2.000 | 1.000 | 0.030 | 0.500 | 8.000 | 0.015 | 0.500 | 2.000 | 4.000 | 256.000 |
| E465-3 | 1 | 0.125 | 0.250 | 0.125 | 1.000 | 0.250 | 0.125 | 0.500 | 0.250 | 1.000 | 0.015 | 0.250 | 1.000 | 1.000 | 251.000 |
| E465-4 | 1 | 4.000 | 0.125 | 8.000 | 1.000 | 0.125 | 0.125 | 0.125 | 0.500 | 0.500 | 0.008 | 0.250 | 0.008 | 2.000 | 251.000 |
| P014 | 1 | 16.000 | 0.250 | 0.250 | 16.000 | 4.000 | 2.000 | 0.030 | 0.500 | 4.000 | 0.015 | 0.250 | 0.008 | 1.000 | 256.000 |
| P015 | 1 | 1.000 | 0.030 | 0.125 | 4.000 | 4.000 | 1.000 | 0.030 | 0.500 | 4.000 | 0.015 | 0.250 | 8.000 | 2.000 | 256.000 |
| P015-2 | 1 | 2.000 | 0.060 | 8.000 | 16.000 | 9.000 | 2.000 | 1.000 | 0.500 | 0.500 | 0.008 | 0.500 | 0.008 | 2.000 | 251.000 |
| P019 | 1 | 0.030 | 0.030 | 0.125 | 0.250 | 0.030 | 0.030 | 0.030 | 0.125 | 4.000 | 0.008 | 0.500 | 0.008 | 0.006 | 251.000 |
| P019-2 | 1 | 2.000 | 0.500 | 2.000 | 16.000 | 0.125 | 0.250 | 4.000 | 1.000 | 0.500 | 0.060 | 0.500 | 0.008 | 0.006 | 251.000 |
| P019-3 | 1 | 0.030 | 0.030 | 0.500 | 0.250 | 0.060 | 0.060 | 0.060 | 1.000 | 1.000 | 0.008 | 0.500 | 0.008 | 0.006 | 251.000 |
| P028 | 1 | 0.500 | 0.030 | 0.125 | 4.000 | 4.000 | 2.000 | 0.030 | 0.500 | 4.000 | 0.015 | 0.250 | 4.000 | 0.500 | 256.000 |
| P028-2 | 1 | 0.125 | 0.125 | 16.000 | 4.000 | 1.000 | 0.500 | 0.500 | 0.250 | 1.000 | 0.008 | 0.250 | 0.008 | 0.250 | 251.000 |
| P040 | 1 | 0.250 | 0.030 | 4.000 | 2.000 | 0.250 | 0.125 | 0.250 | 1.000 | 0.500 | 0.008 | 0.250 | 0.008 | 2.000 | 251.000 |
| P040-2 | 1 | 0.030 | 0.125 | 8.000 | 2.000 | 0.250 | 0.125 | 0.250 | 0.500 | 0.500 | 0.008 | 0.250 | 0.008 | 2.000 | 251.000 |
| P044 | 1 | 0.500 | 0.030 | 0.060 | 4.000 | 4.000 | 1.000 | 0.030 | 1.000 | 4.000 | 0.015 | 0.500 | 8.000 | 4.000 | 256.000 |
| P044-2 | 1 | 0.015 | 0.015 | 0.030 | 0.250 | 0.015 | 0.030 | 0.015 | 0.500 | 0.500 | 0.008 | 0.500 | 0.008 | 0.500 | 128.000 |
| P044-3 | 1 | 0.500 | 0.015 | 8.000 | 4.000 | 1.000 | 1.000 | 0.250 | 1.000 | 0.500 | 0.008 | 0.250 | 0.008 | 2.000 | 251.000 |
| P044-5 | 1 | 0.250 | 0.030 | 8.000 | 8.000 | 1.000 | 1.000 | 0.030 | 1.000 | 0.500 | 0.008 | 0.250 | 0.008 | 2.000 | 251.000 |
| P044-6 | 1 | 0.125 | 0.030 | 8.000 | 4.000 | 0.500 | 1.000 | 0.250 | 0.500 | 1.000 | 0.008 | 0.250 | 0.008 | 2.000 | 251.000 |
| P044-7 | 1 | 0.250 | 0.030 | 8.000 | 8.000 | 1.000 | 1.000 | 0.250 | 0.500 | 1.000 | 0.008 | 0.250 | 0.008 | 2.000 | 251.000 |
| P044-9 | 1 | 0.250 | 0.030 | 8.000 | 4.000 | 1.000 | 1.000 | 0.500 | 1.000 | 1.000 | 0.008 | 0.250 | 0.008 | 2.000 | 251.000 |
| P047 | 1 | 0.500 | 0.030 | 0.030 | 2.000 | 1.000 | 0.500 | 0.060 | 1.000 | 0.500 | 0.015 | 0.500 | 8.000 | 2.000 | 256.000 |
| P047-2 | 1 | 0.125 | 0.030 | 8.000 | 2.000 | 0.500 | 0.500 | 0.060 | 1.000 | 0.500 | 0.008 | 0.250 | 0.008 | 1.000 | 251.000 |
| P047-3 | 1 | 0.500 | 0.030 | 4.000 | 2.000 | 0.500 | 0.500 | 0.125 | 0.500 | 1.000 | 0.008 | 0.500 | 0.008 | 1.000 | 251.000 |
| P049 | 1 | 0.500 | 0.250 | 0.125 | 8.000 | 1.000 | 2.000 | 0.125 | 1.000 | 4.000 | 0.015 | 0.500 | 0.008 | 4.000 | 251.000 |
| P050 | 1 | 0.125 | 0.125 | 0.030 | 1.000 | 0.125 | 0.125 | 0.125 | 1.000 | 4.000 | 0.015 | 0.250 | 0.008 | 1.000 | 251.000 |
| P050-2 | 1 | 0.125 | 0.125 | 8.000 | 1.000 | 0.250 | 0.125 | 0.250 | 1.000 | 1.000 | 0.015 | 0.250 | 0.015 | 1.000 | 251.000 |
| P050-3 | 1 | 0.250 | 0.060 | 8.000 | 2.000 | 0.250 | 0.125 | 0.250 | 1.000 | 1.000 | 0.008 | 0.500 | 0.008 | 2.000 | 251.000 |
| P054-2 | 1 | 0.500 | 0.030 | 8.000 | 2.000 | 1.000 | 0.500 | 0.500 | 0.500 | 0.500 | 0.008 | 0.250 | 0.008 | 2.000 | 251.000 |
| P065 | 1 | 0.250 | 0.030 | 0.125 | 8.000 | 0.500 | 1.000 | 0.500 | 0.500 | 1.000 | 0.015 | 0.500 | 0.008 | 2.000 | 251.000 |
| P065-2 | 1 | 0.500 | 0.030 | 4.000 | 2.000 | 1.000 | 0.500 | 0.500 | 0.500 | 0.500 | 0.008 | 0.250 | 0.008 | 2.000 | 251.000 |
| P065-3 | 1 | 1.000 | 0.030 | 4.000 | 2.000 | 1.000 | 0.500 | 0.500 | 0.500 | 0.500 | 0.008 | 0.250 | 0.008 | 2.000 | 251.000 |
| P067 | 1 | 0.030 | 0.030 | 0.030 | 0.500 | 0.030 | 0.030 | 0.030 | 0.500 | 4.000 | 0.015 | 0.250 | 0.125 | 2.000 | 256.000 |
| P067-2 | 1 | 0.030 | 0.030 | 0.030 | 0.250 | 0.030 | 0.030 | 0.030 | 1.000 | 0.500 | 0.008 | 0.250 | 0.008 | 2.000 | 251.000 |

| Continuation of Table S3 | | | | | | | | | | | | | | | |
|---|---|---|---|---|---|---|---|---|---|---|---|---|---|---|---|
| Strain No. | Clade | BUC | CLO | MIC | FLC | TEC | VRC | ITC | AMB | NYS | AFG | CFG | MFG | 5FC | TRB |
| P070 | 1 | 0.030 | 0.125 | 0.030 | 0.250 | 0.030 | 0.030 | 0.030 | 1.000 | 4.000 | 0.015 | 0.500 | 0.008 | 64.000 | 251.000 |
| P070-2 | 1 | 0.030 | 0.030 | 0.250 | 0.250 | 0.030 | 0.030 | 0.030 | 0.500 | 0.500 | 0.008 | 0.250 | 0.008 | 64.000 | 251.000 |
| P070-3 | 1 | 0.030 | 0.060 | 0.250 | 0.250 | 0.030 | 0.030 | 0.030 | 0.500 | 0.500 | 0.008 | 0.500 | 0.008 | 64.000 | 251.000 |
| P072 | 1 | 0.500 | 0.030 | 0.030 | 4.000 | 1.000 | 1.000 | 0.030 | 1.000 | 4.000 | 0.015 | 0.500 | 2.000 | 1.000 | 256.000 |
| P074 | 1 | 0.250 | 0.125 | 8.000 | 4.000 | 1.000 | 0.500 | 0.250 | 1.000 | 4.000 | 0.008 | 0.500 | 0.008 | 2.000 | 251.000 |
| P074-2 | 1 | 0.500 | 0.030 | 8.000 | 4.000 | 1.000 | 0.500 | 0.250 | 1.000 | 0.500 | 0.008 | 0.500 | 0.008 | 2.000 | 251.000 |
| P074-3 | 1 | 0.125 | 0.125 | 8.000 | 8.000 | 1.000 | 0.500 | 0.500 | 0.500 | 0.500 | 0.008 | 0.500 | 0.008 | 2.000 | 251.000 |
| P079 | 1 | 0.500 | 0.030 | 0.030 | 8.000 | 4.000 | 2.000 | 0.030 | 0.500 | 4.000 | 0.015 | 0.250 | 0.008 | 2.000 | 256.000 |
| P079-2 | 1 | 0.125 | 0.030 | 8.000 | 4.000 | 8.000 | 1.000 | 0.250 | 0.500 | 0.500 | 0.008 | 0.250 | 0.008 | 2.000 | 256.000 |
| P085 | 1 | 0.250 | 0.030 | 0.030 | 1.000 | 0.030 | 0.030 | 0.030 | 1.000 | 4.000 | 0.015 | 0.125 | 0.500 | 2.000 | 256.000 |
| P085-2 | 1 | 0.030 | 0.030 | 1.000 | 0.500 | 0.125 | 0.030 | 0.030 | 1.000 | 0.500 | 0.008 | 0.250 | 0.008 | 1.000 | 251.000 |
| P107 | 1 | 0.030 | 0.125 | 4.000 | 1.000 | 0.125 | 0.125 | 0.125 | 0.125 | 2.000 | 0.008 | 0.500 | 0.008 | 1.000 | 251.000 |
| P107-2 | 1 | 0.250 | 0.125 | 8.000 | 1.000 | 0.125 | 0.125 | 0.250 | 0.500 | 0.500 | 0.008 | 0.500 | 0.008 | 2.000 | 251.000 |
| P121 | 1 | 0.250 | 0.030 | 0.030 | 1.000 | 0.500 | 0.250 | 0.030 | 0.500 | 4.000 | 0.015 | 0.060 | 1.000 | 1.000 | 256.000 |
| P121-2 | 1 | 0.125 | 0.060 | 8.000 | 2.000 | 0.250 | 0.250 | 0.125 | 0.500 | 0.500 | 0.008 | 0.250 | 0.008 | 1.000 | 256.000 |
| P122 | 1 | 0.030 | 0.030 | 0.030 | 0.125 | 0.030 | 0.030 | 0.030 | 0.500 | 4.000 | 0.015 | 0.250 | 0.030 | 128.000 | 128.000 |
| P122-4 | 1 | 0.030 | 0.060 | 0.500 | 0.125 | 0.030 | 0.030 | 0.030 | 0.500 | 0.500 | 0.008 | 0.250 | 0.008 | 64.000 | 256.000 |
| P128 | 1 | 4.000 | 0.125 | 0.250 | 16.000 | 8.000 | 4.000 | 0.030 | 0.500 | 2.000 | 0.015 | 0.250 | 4.000 | 2.000 | 256.000 |
| P133 | 1 | 0.125 | 0.030 | 0.030 | 1.000 | 0.500 | 0.500 | 0.030 | 1.000 | 4.000 | 0.030 | 0.500 | 4.000 | 1.000 | 4.000 |
| P135 | 1 | 0.030 | 0.030 | 0.030 | 0.250 | 0.030 | 0.030 | 0.030 | 0.500 | 4.000 | 0.015 | 0.500 | 0.125 | 1.000 | 256.000 |
| P135-2 | 1 | 0.030 | 0.125 | 1.000 | 0.250 | 0.030 | 0.030 | 0.125 | 1.000 | 1.000 | 0.008 | 0.250 | 0.008 | 1.000 | 251.000 |
| P137 | 1 | 0.125 | 0.060 | 0.030 | 2.000 | 1.000 | 0.500 | 0.030 | 0.060 | 8.000 | 0.015 | 0.500 | 4.000 | 2.000 | 8.000 |
| P137-2 | 1 | 0.125 | 0.030 | 8.000 | 8.000 | 0.500 | 0.500 | 0.500 | 1.000 | 1.000 | 0.008 | 0.250 | 0.008 | 2.000 | 251.000 |
| P139 | 1 | 0.250 | 0.060 | 0.060 | 2.000 | 1.000 | 1.000 | 0.030 | 0.500 | 4.000 | 0.015 | 0.005 | 0.008 | 1.000 | 128.000 |
| P139-2 | 1 | 0.125 | 0.030 | 8.000 | 4.000 | 1.000 | 1.000 | 0.250 | 0.500 | 0.500 | 0.015 | 0.250 | 0.008 | 2.000 | 251.000 |
| P144 | 1 | 0.125 | 0.030 | 0.030 | 2.000 | 1.000 | 0.500 | 0.030 | 0.500 | 4.000 | 0.015 | 0.005 | 0.008 | 2.000 | 32.000 |
| P144-2 | 1 | 0.125 | 0.030 | 8.000 | 4.000 | 8.000 | 1.000 | 0.250 | 0.500 | 0.500 | 0.008 | 0.250 | 0.008 | 2.000 | 251.000 |
| T001 | 1 | 0.125 | 0.250 | 0.030 | 2.000 | 0.500 | 0.500 | 0.060 | 0.250 | 0.500 | 0.015 | 0.250 | 2.000 | 1.000 | 256.000 |
| T006 | 1 | 2.000 | 0.125 | 0.030 | 1.000 | 2.000 | 2.000 | 0.500 | 0.500 | 0.500 | 0.015 | 0.125 | 2.000 | 1.000 | 256.000 |
| T010 | 1 | 2.000 | 0.030 | 0.250 | 2.000 | 1.000 | 0.500 | 0.030 | 0.250 | 4.000 | 0.015 | 0.250 | 2.000 | 1.000 | 256.000 |
| T017 | 1 | 1.000 | 0.030 | 0.030 | 1.000 | 0.250 | 0.060 | 0.030 | 0.250 | 4.000 | 0.015 | 0.250 | 4.000 | 1.000 | 256.000 |
| T042 | 1 | 2.000 | 0.125 | 0.030 | 1.000 | 1.000 | 0.250 | 0.500 | 0.250 | 0.250 | 0.015 | 0.125 | 1.000 | 1.000 | 256.000 |
| T069 | 1 | 2.000 | 0.030 | 0.060 | 4.000 | 1.000 | 1.000 | 0.060 | 0.500 | 0.500 | 0.015 | 0.250 | 4.000 | 1.000 | 251.000 |
| T093 | 1 | 1.000 | 0.030 | 0.060 | 1.000 | 0.500 | 0.060 | 0.060 | 0.250 | 4.000 | 0.015 | 0.250 | 2.000 | 1.000 | 256.000 |
| T105 | 1 | 2.000 | 0.030 | 0.060 | 1.000 | 1.000 | 1.000 | 0.060 | 0.500 | 0.500 | 0.015 | 0.250 | 4.000 | 1.000 | 256.000 |
| T111 | 1 | 2.000 | 0.030 | 0.250 | 2.000 | 2.000 | 0.500 | 0.250 | 0.250 | 4.000 | 0.015 | 0.250 | 2.000 | 1.000 | 256.000 |
| T147 | 1 | 0.060 | 0.030 | 0.500 | 1.000 | 1.000 | 4.000 | 0.500 | 0.500 | 0.500 | 0.015 | 0.250 | 0.008 | 1.000 | 256.000 |
| T150 | 1 | 0.250 | 1.000 | 0.030 | 1.000 | 1.000 | 0.500 | 2.000 | 0.250 | 0.500 | 0.030 | 1.000 | 2.000 | 16.000 | 256.000 |
| T343 | 1 | 0.125 | 0.060 | 0.030 | 2.000 | 1.000 | 1.000 | 0.125 | 0.500 | 0.500 | 0.015 | 0.250 | 0.015 | 2.000 | 256.000 |
| 8574 | 3 | 0.030 | 0.030 | 0.015 | 0.060 | 0.030 | 0.030 | 0.030 | 0.250 | 0.250 | 0.008 | 0.250 | 0.030 | 0.500 | 256.000 |
| 8906 | 3 | 0.030 | 0.030 | 0.015 | 0.060 | 0.060 | 0.030 | 0.030 | 0.125 | 0.500 | 0.008 | 0.250 | 0.030 | 0.250 | 256.000 |
| 9244 | 3 | 0.030 | 0.030 | 0.015 | 0.060 | 0.060 | 0.030 | 0.030 | 0.125 | 0.250 | 0.008 | 0.250 | 0.015 | 0.250 | 256.000 |
| P060 | 3 | 0.060 | 0.030 | 0.030 | 2.000 | 0.030 | 32.000 | 0.030 | 0.500 | 4.000 | 0.015 | 0.005 | 0.125 | 0.250 | 256.000 |
| P060-2 | 3 | 0.030 | 0.060 | 0.030 | 0.125 | 0.030 | 0.030 | 0.030 | 0.250 | 0.500 | 0.008 | 0.500 | 0.008 | 0.125 | 128.000 |
| T194 | 3 | 0.030 | 0.030 | 0.030 | 0.125 | 0.030 | 0.030 | 0.030 | 0.060 | 0.250 | 0.015 | 0.250 | 0.015 | 0.125 | 256.000 |
| 8751 | 4 | 0.030 | 0.030 | 0.125 | 0.500 | 0.030 | 0.030 | 0.030 | 0.060 | 0.500 | 0.015 | 0.030 | 0.015 | 0.125 | 256.000 |
| P145 | 4 | 0.030 | 0.030 | 0.030 | 0.250 | 0.030 | 0.030 | 0.030 | 1.000 | 4.000 | 0.015 | 0.005 | 0.060 | 0.125 | 128.000 |

| Continuation of Table S3 | | | | | | | | | | | | | | | |
|---|---|---|---|---|---|---|---|---|---|---|---|---|---|---|---|
| Strain No. | Clade | BUC | CLO | MIC | FLC | TEC | VRC | ITC | AMB | NYS | AFG | CFG | MFG | 5FC | TRB |
| T112 | 4 | 0.030 | 0.030 | 0.015 | 0.060 | 0.030 | 0.030 | 0.030 | 0.060 | 0.250 | 0.008 | 0.250 | 0.015 | 0.250 | 128.000 |
| T188 | 4 | 0.030 | 0.030 | 0.030 | 0.125 | 0.030 | 0.030 | 0.030 | 0.030 | 0.250 | 0.015 | 0.250 | 0.015 | 0.125 | 256.000 |
| T320 | 4 | 0.125 | 0.015 | 0.015 | 0.125 | 0.030 | 0.030 | 0.030 | 0.125 | 0.250 | 0.008 | 0.500 | 0.030 | 0.250 | 256.000 |
| E101 | 6 | 0.015 | 0.030 | 0.015 | 0.060 | 0.030 | 0.030 | 0.030 | 0.125 | 0.500 | 0.008 | 0.250 | 0.030 | 0.250 | 256.000 |
| E206 | 6 | 0.030 | 0.030 | 0.030 | 1.000 | 0.030 | 0.030 | 0.030 | 0.060 | 0.250 | 0.015 | 0.500 | 1.000 | 0.500 | 128.000 |
| E070 | 6 | 0.030 | 0.030 | 0.030 | 0.125 | 0.030 | 0.030 | 0.030 | 0.030 | 0.250 | 0.015 | 0.250 | 0.015 | 0.125 | 256.000 |
| 1035 | 8 | 0.030 | 0.015 | 0.030 | 0.125 | 0.030 | 0.030 | 0.030 | 0.060 | 2.000 | 0.015 | 0.250 | 0.125 | 0.125 | 256.000 |
| 2252 | 8 | 4.000 | 0.030 | 0.125 | 0.500 | 0.030 | 0.030 | 0.030 | 0.060 | 0.500 | 0.015 | 0.125 | 2.000 | 0.250 | 256.000 |
| 2284 | 8 | 0.030 | 0.030 | 0.060 | 0.125 | 0.030 | 0.030 | 0.030 | 0.060 | 0.500 | 0.015 | 0.250 | 0.015 | 0.250 | 256.000 |
| 5028 | 8 | 0.030 | 0.030 | 0.060 | 0.125 | 0.030 | 0.030 | 0.030 | 0.250 | 4.000 | 0.015 | 0.250 | 0.015 | 0.125 | 256.000 |
| 5079 | 8 | 0.030 | 0.015 | 0.060 | 0.250 | 0.015 | 0.030 | 0.030 | 0.060 | 1.000 | 0.008 | 0.250 | 0.015 | 0.250 | 128.000 |
| 5132 | 8 | 0.030 | 0.030 | 0.060 | 0.125 | 0.030 | 0.030 | 0.030 | 0.250 | 0.500 | 0.015 | 0.250 | 0.015 | 0.125 | 256.000 |
| 8198 | 8 | 0.030 | 0.030 | 0.060 | 0.125 | 0.030 | 0.030 | 0.030 | 0.500 | 0.500 | 0.015 | 0.250 | 0.015 | 0.125 | 256.000 |
| 8471 | 8 | 0.030 | 0.030 | 0.015 | 0.060 | 0.060 | 0.030 | 0.030 | 0.250 | 1.000 | 0.008 | 0.250 | 0.060 | 0.250 | 256.000 |
| 8807 | 8 | 0.125 | 0.030 | 0.125 | 0.250 | 0.030 | 0.030 | 0.030 | 0.250 | 0.500 | 0.015 | 0.500 | 0.030 | 0.250 | 128.000 |
| 2522763 | 8 | 0.125 | 0.030 | 0.060 | 0.250 | 0.250 | 0.060 | 0.060 | 0.250 | 0.500 | 0.015 | 0.500 | 0.060 | 0.125 | 256.000 |
| 9053 | 8 | 0.030 | 0.015 | 0.030 | 0.125 | 0.030 | 0.030 | 0.030 | 0.060 | 2.000 | 0.015 | 0.500 | 0.015 | 0.500 | 256.000 |
| E442 | 8 | 0.030 | 0.030 | 0.030 | 0.125 | 0.060 | 0.030 | 0.030 | 0.060 | 0.250 | 0.015 | 0.250 | 0.015 | 0.125 | 256.000 |
| P005 | 8 | 0.030 | 0.030 | 0.030 | 0.250 | 0.030 | 0.030 | 0.030 | 1.000 | 4.000 | 0.015 | 0.250 | 0.125 | 0.250 | 32.000 |
| P005-3 | 8 | 0.030 | 0.030 | 0.030 | 0.125 | 0.030 | 0.030 | 0.030 | 0.500 | 1.000 | 0.008 | 0.500 | 0.008 | 0.250 | 251.000 |
| P005-4 | 8 | 0.030 | 0.030 | 4.000 | 0.125 | 0.030 | 0.030 | 0.030 | 1.000 | 0.500 | 0.008 | 0.500 | 0.008 | 0.250 | 251.000 |
| P134 | 8 | 0.030 | 0.125 | 0.030 | 0.500 | 0.030 | 0.030 | 0.030 | 0.500 | 4.000 | 0.030 | 0.500 | 0.008 | 0.250 | 251.000 |
| T100 | 8 | 0.030 | 0.030 | 0.030 | 0.125 | 0.030 | 0.030 | 0.030 | 0.125 | 0.500 | 0.015 | 0.015 | 0.015 | 0.125 | 128.000 |
| T104 | 8 | 0.030 | 0.030 | 0.030 | 0.125 | 0.030 | 0.030 | 0.030 | 0.060 | 0.500 | 0.015 | 0.250 | 0.015 | 0.125 | 16.000 |
| T189 | 8 | 0.030 | 0.015 | 0.060 | 0.500 | 0.030 | 0.030 | 0.030 | 0.060 | 1.000 | 0.015 | 0.250 | 0.015 | 0.125 | 256.000 |
| T321 | 8 | 0.030 | 0.030 | 0.015 | 0.060 | 0.030 | 0.030 | 0.030 | 0.125 | 0.250 | 0.008 | 0.250 | 0.060 | 0.125 | 128.000 |
| T476 | 8 | 0.030 | 0.030 | 0.030 | 0.250 | 0.030 | 0.060 | 0.030 | 0.060 | 0.500 | 0.015 | 0.500 | 0.015 | 0.125 | 256.000 |
| E229 | 9 | 0.030 | 0.030 | 0.030 | 0.250 | 0.030 | 0.030 | 0.030 | 0.060 | 0.500 | 0.015 | 0.500 | 0.015 | 0.125 | 128.000 |
| 1112 | 11 | 0.030 | 0.030 | 0.030 | 0.250 | 0.030 | 0.030 | 0.030 | 0.125 | 0.500 | 0.015 | 0.250 | 0.015 | 0.125 | 256.000 |
| 8182 | 11 | 0.030 | 0.015 | 0.060 | 2.000 | 0.500 | 0.250 | 0.060 | 0.060 | 1.000 | 0.008 | 0.500 | 0.500 | 0.250 | 256.000 |
| 8232 | 11 | 0.030 | 0.030 | 0.030 | 0.125 | 0.030 | 0.030 | 0.030 | 0.030 | 0.250 | 0.015 | 0.250 | 0.015 | 0.125 | 256.000 |
| 8311 | 11 | 0.030 | 0.030 | 0.125 | 1.000 | 0.030 | 0.030 | 0.030 | 0.500 | 0.500 | 0.015 | 0.250 | 0.125 | 0.250 | 256.000 |
| 2341661 | 11 | 0.030 | 0.030 | 0.030 | 0.125 | 0.125 | 0.030 | 0.030 | 1.000 | 0.500 | 0.015 | 0.250 | 0.015 | 0.125 | 256.000 |
| P152 | 11 | 0.500 | 0.060 | 0.030 | 0.250 | 0.030 | 0.030 | 0.030 | 0.125 | 4.000 | 0.008 | 0.250 | 0.008 | 0.500 | 251.000 |
| P152-2 | 11 | 0.030 | 0.125 | 0.500 | 0.500 | 0.030 | 0.030 | 0.125 | 0.500 | 1.000 | 0.008 | 0.250 | 0.008 | 0.500 | 251.000 |
| 9427 | 12 | 0.500 | 0.125 | 0.015 | 0.125 | 0.030 | 0.030 | 0.030 | 0.125 | 0.250 | 0.008 | 0.500 | 0.008 | 0.500 | 256.000 |
| E402 | 12 | 0.060 | 0.030 | 0.015 | 0.060 | 0.125 | 0.030 | 0.030 | 0.500 | 0.500 | 0.008 | 0.250 | 0.015 | 5.000 | 128.000 |
| F104 | 12 | 0.060 | 0.030 | 0.015 | 0.060 | 0.060 | 0.060 | 0.125 | 0.500 | 0.500 | 0.008 | 0.250 | 0.500 | 1.000 | 256.000 |
| 1021 | 14 | 0.030 | 0.030 | 0.015 | 0.060 | 0.030 | 0.030 | 0.030 | 0.060 | 0.500 | 0.008 | 0.250 | 0.030 | 0.125 | 128.000 |
| 1034 | 14 | 0.030 | 0.015 | 0.060 | 0.500 | 0.030 | 0.030 | 0.030 | 0.500 | 2.000 | 0.008 | 0.250 | 0.015 | 0.250 | 256.000 |
| 2206 | 14 | 0.030 | 0.015 | 0.250 | 0.250 | 0.030 | 0.030 | 0.030 | 0.060 | 2.000 | 0.008 | 0.125 | 0.015 | 0.250 | 256.000 |
| 8134 | 14 | 0.030 | 0.015 | 0.500 | 0.500 | 1.000 | 0.250 | 0.030 | 0.060 | 2.000 | 0.008 | 0.030 | 0.015 | 0.250 | 256.000 |
| 8149 | 14 | 0.500 | 0.030 | 0.015 | 0.060 | 4.000 | 0.500 | 0.030 | 0.125 | 0.250 | 0.008 | 0.125 | 0.030 | 0.006 | 32.000 |
| 8221 | 14 | 0.030 | 0.030 | 0.015 | 0.125 | 0.030 | 0.030 | 0.030 | 0.125 | 0.500 | 0.008 | 0.250 | 0.015 | 0.125 | 128.000 |
| 8347 | 14 | 0.030 | 0.015 | 0.125 | 1.000 | 0.030 | 0.030 | 0.030 | 0.125 | 0.250 | 0.008 | 0.250 | 0.015 | 0.125 | 256.000 |
| 8437 | 14 | 0.030 | 0.030 | 0.015 | 0.060 | 0.030 | 0.030 | 0.030 | 0.250 | 0.500 | 0.008 | 0.250 | 0.015 | 0.125 | 128.000 |
| 8737 | 14 | 0.030 | 0.030 | 0.030 | 0.125 | 0.030 | 0.030 | 0.030 | 0.060 | 0.250 | 0.015 | 0.250 | 0.015 | 1.000 | 256.000 |

| Strain No. | Clade | BUC | CLO | MIC | FLC | TEC | VRC | ITC | AMB | NYS | AFG | CFG | MFG | 5FC | TRB |
|---|---|---|---|---|---|---|---|---|---|---|---|---|---|---|---|
| Continuation of Table S3 | | | | | | | | | | | | | | | |
| 8896 | 14 | 0.030 | 0.030 | 0.030 | 0.125 | 0.030 | 0.030 | 0.250 | 0.060 | 0.030 | 0.015 | 0.250 | 0.015 | 0.125 | 128.000 |
| E066 | 14 | 0.030 | 0.030 | 0.015 | 0.125 | 0.030 | 0.030 | 0.030 | 0.125 | 0.500 | 0.008 | 0.500 | 0.030 | 0.250 | 128.000 |
| E210 | 14 | 0.030 | 0.015 | 0.015 | 0.060 | 0.030 | 0.030 | 0.030 | 0.125 | 0.500 | 0.008 | 0.250 | 0.015 | 0.125 | 8.000 |
| E424 | 14 | 0.015 | 0.015 | 0.015 | 0.060 | 0.030 | 0.030 | 0.030 | 0.125 | 0.500 | 0.008 | 0.250 | 0.030 | 0.125 | 128.000 |
| E424-3 | 14 | 0.030 | 0.030 | 0.060 | 0.250 | 0.030 | 0.030 | 0.030 | 0.125 | 8.000 | 0.015 | 0.500 | 0.015 | 0.250 | 128.000 |
| E424-4 | 14 | 0.060 | 0.030 | 0.060 | 0.250 | 0.030 | 0.030 | 0.030 | 0.250 | 0.500 | 0.015 | 0.005 | 0.060 | 0.125 | 32.000 |
| T118 | 14 | 0.060 | 0.030 | 0.015 | 0.060 | 0.060 | 0.030 | 0.030 | 0.125 | 0.500 | 0.008 | 0.250 | 0.015 | 0.250 | 128.000 |
| T341 | 14 | 0.030 | 0.030 | 0.015 | 0.060 | 0.030 | 0.030 | 0.030 | 0.500 | 0.500 | 0.008 | 0.250 | 0.015 | 0.125 | 256.000 |
| T499 | 14 | 0.125 | 0.015 | 0.015 | 0.125 | 0.060 | 0.030 | 0.030 | 0.125 | 0.250 | 0.008 | 0.250 | 0.015 | 0.250 | 128.000 |
| 5250 | 15 | 0.060 | 0.030 | 0.060 | 0.250 | 0.060 | 0.030 | 0.030 | 1.000 | 0.500 | 0.008 | 0.060 | 0.008 | 0.006 | 251.000 |
| 5257 | 15 | 0.030 | 0.015 | 0.250 | 0.500 | 0.030 | 32.000 | 0.030 | 0.060 | 0.250 | 0.008 | 0.015 | 0.015 | 0.250 | 256.000 |
| 9457 | 15 | 0.125 | 0.030 | 0.030 | 0.250 | 0.030 | 0.030 | 0.030 | 0.500 | 0.500 | 0.015 | 0.250 | 0.125 | 64.000 | 256.000 |
| E161 | 15 | 0.030 | 0.030 | 0.015 | 0.060 | 0.030 | 0.030 | 0.030 | 0.250 | 0.500 | 0.008 | 0.250 | 0.030 | 0.125 | 256.000 |
| E208 | 15 | 0.030 | 0.030 | 0.015 | 0.060 | 0.030 | 0.030 | 0.030 | 0.250 | 0.500 | 0.008 | 0.250 | 0.015 | 5.000 | 64.000 |
| T062 | 15 | 0.030 | 0.060 | 0.015 | 0.060 | 0.030 | 0.030 | 0.030 | 0.250 | 0.500 | 0.008 | 0.250 | 0.015 | 0.125 | 32.000 |
| T276 | 15 | 0.500 | 0.015 | 0.015 | 0.250 | 0.030 | 0.030 | 0.030 | 0.060 | 0.250 | 0.008 | 0.500 | 0.250 | 0.250 | 256.000 |
| T284 | 15 | 0.030 | 0.030 | 0.015 | 0.500 | 0.250 | 0.060 | 0.125 | 0.500 | 0.500 | 0.060 | 0.500 | 0.015 | 0.125 | 256.000 |
| T295 | 15 | 0.030 | 0.030 | 0.015 | 0.060 | 0.030 | 0.030 | 0.030 | 0.250 | 0.500 | 0.008 | 0.250 | 0.015 | 0.125 | 256.000 |
| 1131 | 17 | 0.030 | 0.030 | 0.015 | 0.250 | 0.030 | 0.030 | 0.030 | 0.250 | 0.500 | 0.008 | 0.250 | 0.015 | 0.125 | 128.000 |
| 5049 | 17 | 0.125 | 0.250 | 0.015 | 0.250 | 0.500 | 0.060 | 0.060 | 0.250 | 0.500 | 0.008 | 0.500 | 0.060 | 1.000 | 256.000 |
| 8115 | 17 | 0.030 | 0.030 | 0.015 | 0.060 | 0.030 | 0.030 | 0.030 | 0.500 | 0.500 | 0.008 | 0.250 | 0.250 | 0.500 | 256.000 |
| 8564 | 17 | 2.000 | 0.030 | 0.015 | 0.060 | 0.030 | 0.060 | 0.060 | 0.125 | 0.250 | 0.008 | 0.250 | 0.030 | 0.006 | 256.000 |
| E047 | 17 | 0.030 | 0.030 | 0.015 | 0.125 | 0.060 | 0.060 | 0.030 | 0.250 | 0.500 | 0.008 | 0.250 | 0.030 | 0.500 | 128.000 |
| E107 | 17 | 0.060 | 0.030 | 0.015 | 0.060 | 0.060 | 0.030 | 0.030 | 0.125 | 0.250 | 0.008 | 0.250 | 0.015 | 0.250 | 256.000 |
| E140 | 17 | 2.000 | 0.015 | 0.250 | 0.060 | 0.060 | 0.060 | 0.030 | 0.060 | 0.500 | 0.008 | 0.060 | 0.030 | 0.250 | 256.000 |
| E331 | 17 | 0.030 | 0.030 | 0.060 | 0.125 | 0.030 | 0.030 | 0.030 | 0.500 | 0.500 | 0.015 | 0.250 | 0.015 | 0.125 | 256.000 |
| T030 | 17 | 0.500 | 0.125 | 0.015 | 0.250 | 0.030 | 0.030 | 0.030 | 0.125 | 0.250 | 0.008 | 0.500 | 0.250 | 0.250 | 256.000 |
| T068 | 17 | 0.030 | 0.015 | 0.060 | 0.500 | 0.030 | 0.030 | 0.030 | 0.060 | 1.000 | 0.008 | 0.250 | 0.015 | 0.250 | 128.000 |
| T349 | 17 | 0.030 | 0.030 | 0.015 | 0.060 | 0.030 | 0.030 | 0.030 | 0.060 | 0.250 | 0.008 | 0.250 | 0.015 | 0.250 | 256.000 |
| 8558 | 17 | 0.125 | 0.030 | 0.125 | 0.250 | 0.030 | 0.030 | 0.030 | 0.250 | 8.000 | 0.015 | 0.250 | 0.060 | 0.250 | 32.000 |
| 2431818 | 17 | 0.030 | 0.030 | 0.060 | 0.250 | 0.030 | 0.030 | 0.030 | 0.060 | 0.500 | 0.015 | 0.250 | 0.008 | 0.250 | 16.000 |
| 2276618 | 101 | 0.030 | 0.030 | 0.060 | 0.250 | 0.030 | 0.030 | 0.030 | 0.500 | 8.000 | 0.015 | 0.250 | 0.250 | 0.250 | 128.000 |
| T129 | 101 | 0.030 | 0.030 | 0.015 | 0.060 | 0.030 | 0.030 | 0.030 | 0.060 | 0.500 | 0.008 | 0.250 | 0.015 | 0.250 | 128.000 |
| T164 | 101 | 0.030 | 0.030 | 0.015 | 0.125 | 0.060 | 0.030 | 0.030 | 1.000 | 1.000 | 0.008 | 0.500 | 0.060 | 0.250 | 128.000 |
| E165-11 | 102 | 0.500 | 0.030 | 0.250 | 0.500 | 0.030 | 0.250 | 0.030 | 0.125 | 0.500 | 0.015 | 0.500 | 0.008 | 0.250 | 0.030 |
| E165-8 | 102 | 0.030 | 0.030 | 0.250 | 1.000 | 0.030 | 0.030 | 0.030 | 0.500 | 0.500 | 0.015 | 0.500 | 0.125 | 0.250 | 128.000 |
| E165-9 | 102 | 0.500 | 0.030 | 0.125 | 16.000 | 0.030 | 0.030 | 0.030 | 0.250 | 0.500 | 0.015 | 0.250 | 0.008 | 0.250 | 256.000 |
| T314 | 102 | 0.500 | 0.015 | 0.250 | 0.500 | 0.060 | 16.000 | 0.030 | 0.060 | 0.500 | 0.008 | 0.030 | 0.125 | 0.125 | 256.000 |
| T405 | 103 | 0.030 | 0.030 | 0.030 | 0.125 | 0.030 | 0.030 | 0.030 | 0.060 | 0.500 | 0.015 | 0.250 | 0.015 | 0.125 | 256.000 |
| T160 | 103 | 0.030 | 0.030 | 0.030 | 0.125 | 0.030 | 0.030 | 0.030 | 0.060 | 0.500 | 0.015 | 0.250 | 0.015 | 0.125 | 256.000 |

End of Table S3

**Table S4.** Value of the extracellular enzymatic activity of different MLST genotypes of *C. albicans*.

| Clade | Phospholipase | Esterase | Haemolysis |
|---|---|---|---|
| Clade1 | 0.592±0.069 | 0.564 ±0.061 | 0.407 ±0.064 |
| Clade3 | 0.575±0.044 | 0.363 ±0.078 | 0.356 ±0.045 |
| Clade4 | 0.533±0.040 | 0.409 ±0.066 | 0.356 ±0.045 |
| Clade6 | 0.432±0.011 | 0.387±0.012 | 0.321 ±0.011 |
| Clade8 | 0.622±0.036 | 0.509 ±0.057 | 0.365 ±0.066 |
| Clade9 | 0.591 | 0.479 | 0.313 |
| Clade11 | 0.572±0.046 | 0.475 ±0.114 | 0.391 ±0.079 |
| Clade12 | 0.549±0.062 | 0.355 ±0.045 | 0.317 ±0.040 |
| Clade14 | 0.570±0.061 | 0.361 ±0.049 | 0.323 ±0.023 |
| Clade15 | 0.511±0.081 | 0.372 ±0.040 | 0.334 ±0.060 |
| Clade17 | 0.571±0.055 | 0.353 ±0.031 | 0.346 ±0.052 |
| Clade100 | 0.576±0.072 | 0.382± 0.053 | 0.314 ±0.028 |
| Clade101 | 0.531±0.060 | 0.368 ±0.000 | 0.306 ±0.039 |
| Clade 102 | 0.581 | 0.350 | 0.333 |
| Clade 103 | 0.591±0.012 | 0.479 ±0.000 | 0.313 ±0.021 |
| Total | 0.575±0.068 | 0.464± 0.106 | 0.366 ±0.064 |
